# CO_2_ Reduction to Methane and Ethylene on a Single-Atom Catalyst: A Grand Canonical Quantum Mechanics Study

**DOI:** 10.1021/jacs.3c05650

**Published:** 2023-09-20

**Authors:** Silvio Osella, William A. Goddard III

**Affiliations:** † Chemical and Biological Systems Simulation Lab, Centre of New Technologies, University of Warsaw, Banacha 2C, 02-097 Warsaw, Poland; ‡ Materials and Process Simulation Center (MSC), California Institute of Technology, MC 139-74, Pasadena, California 91125, United States

## Abstract

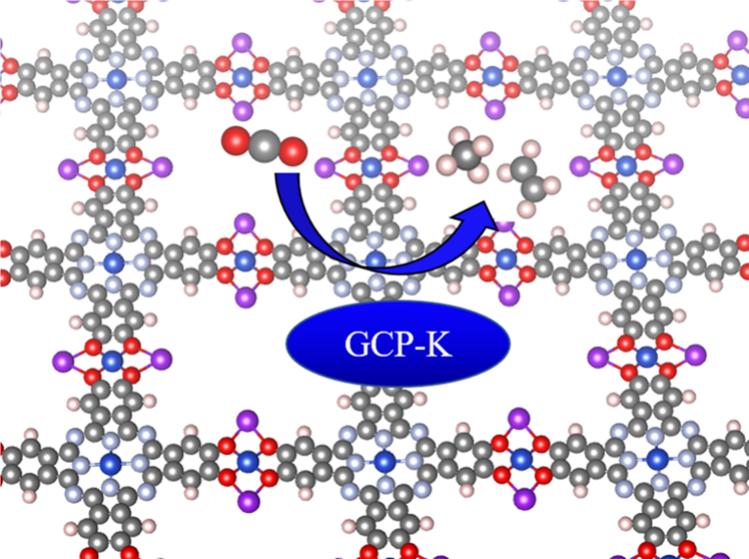

In recent years, two-dimensional metal–organic frameworks (2D MOF) have attracted great interest for their ease of synthesis and for their catalytic activities and semiconducting properties. The appeal of these materials is that they are layered and easily exfoliated to obtain a monolayer (or few layer) material with interesting optoelectronic properties. Moreover, they have great potential for CO_2_ reduction to obtain solar fuels with more than one carbon atom, such as ethylene and ethanol, in addition to methane and methanol. In this paper, we explore how a particular class of 2D MOF based on a phthalocyanine core provides the reactive center for the production of ethylene and ethanol. We examine the reaction mechanism using the new grand canonical potential kinetics (GCP-K) or grand canonical quantum mechanics (GC-QM) computational methodology, which obtains reaction rates at constant applied potential to compare directly with experimental results (rather than at constant electrons as in standard QM). We explain the reaction mechanism underlying the formation of methane and ethylene. Here, the key reaction step is direct coupling of CO into CHO, without the usual rate-determining CO–CO dimerization step observed on Cu metal surfaces. Indeed, the 2D MOF behaves like a single-atom catalyst.

## 1 Introduction

Electrocatalytic (EC) conversion of CO_2_ into valuable chemicals such as methane, ethylene, and ethanol is the most promising for both environmental and energy purposes. Despite decades of research on CO_2_ reduction reaction (CO_2_RR) to hydrocarbon products, the best electrocatalyst remains metallic copper, albeit with low faradic efficiency (FE) for any particular product and low durability due to oxygen depletion after 15 h.^1,2^ Recent experimental and computational studies show a correlation between the metal surface facet [e.g., Cu(100) or Cu(111)] and the FE,^3−6^ as well as enhancement in FE when the metal is coated with an appropriate microenvironment.^7^ But current technology has not yet reached commercial application, primarily because the selectivity (faradic efficiency) remains too low, with the reaction kinetics too slow for ethylene or ethanol. To obtain a high FE for these products, it is essential to promote C–C dimerization, which is generally the rate-determining step for C2 production. This C–C coupling step is facilitated on the Cu surface, leading to high yield and selectivity toward multiple carbon products. In contrast to semiconductor materials with only one single atom at the catalytic center, C–C dimerization is generally considered, which is why we focus here on low-dimensional metal–organic frameworks (MOFs).

The majority of traditional MOFs are electrical insulators, which limits their use in multifunctional electronic devices.^8^ However, recent studies show the ability of Cu-doped three-dimensional (3D) MOF performing electrocatalytic CO_2_RR to obtain gas products (such as formaldehyde and ethylene)^9,10^ and liquid products (such as methanol and ethanol) with good FE and high selectivity.^11−14^ Moreover, their assembly to form layered architectures is a promising strategy to obtain ethylene and ethanol in relatively high FE.^15,16^ Yet, the intrinsic advantage of the two-dimensional conjugated MOFs (2D MOFs) is their nature as semiconductors. These layered MOFs often consist of a conjugated building block core (e.g., triphenylene or phthalocyanine) connected by either hexagonal or square-planar linkages with high in-plane conjugation and weak out-of-plane van der Waals interactions. This extended π-conjugation in the basal plane allows delocalization of charge carriers within the network, which is beneficial to high mobility and conductivity.^17^

In addition to the well-defined active sites, 2D MOFs possess unique chemical and physical features such as high stability, electrochemical activity, photoactivity, tailorable band gaps, and superior electrical conductivity.^18−20^ Moreover, their catalytic activity can be extremely high and suitable for the production of different chemicals by fine-tuning the metal centers and linkers. Despite the progress of 2D MOFs for electrocatalysis, many critical challenges remain in the design and synthesis of advanced 2D MOFs with an optimal structure for electrocatalytic activity for particular catalytic reactions for practical applications.^21^

**Figure 1:**
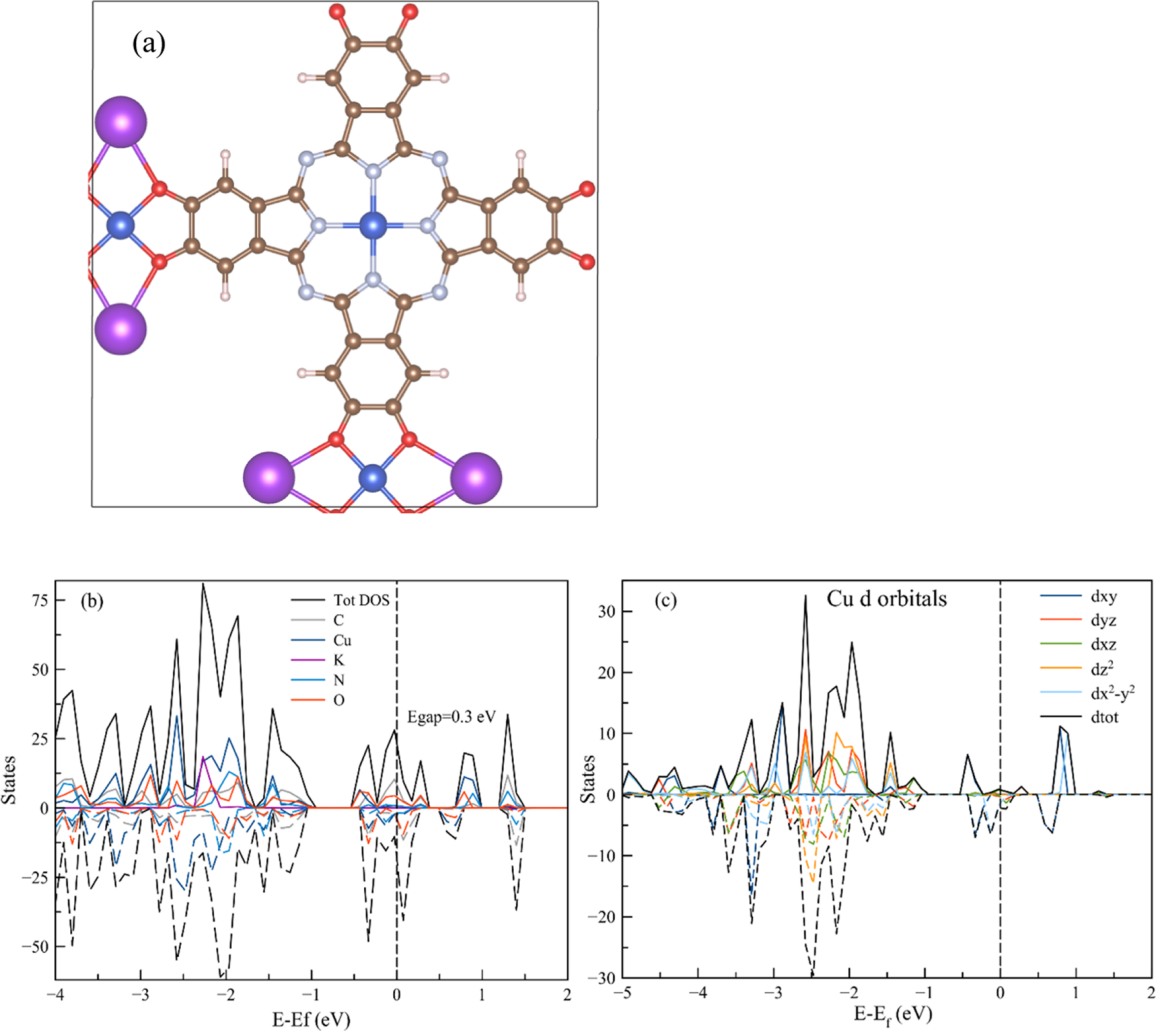
(a) Structure of the 2D MOF model catalyst in this study. (b, c) Total and projected density of states analysis of the MOF cell and for the d orbitals of the Cu atoms. Color code: brown—carbon; white—hydrogen; blue—copper; red—oxygen; cyan—nitrogen; purple—potassium.

Among the various synthesized 2D MOFs, the MOF based on linkers containing a phthalocyanine core connecting to pairs of CuO_4_ nodes (PcCu, Figure 1a) has already shown impressive performance for producing solar fuels depending on the nature of the metal centers. As an example, with the same structure

- using copper ions in both the phthalocyanine and the linker centers, the CO_2_RR leads to ethylene with a faradic effficiency (FE) up to 50% (the highest reported to date).^22^
- using zinc in the linker leads to CO as the main product (FE = 88%).^23^
- using cobalt leads to the oxygen reduction reaction (ORR) important for hydrogen fuel cells.^24^
- replacing all coppers with nickel ions leads to the oxygen evolution reaction (OER) instead of CO_2_RR.^25^

Despite the high versatility of this single 2D MOF structure to allow different products, the reaction mechanism underlying its catalytic activity and the role of the metal at the center and on the linkers remain obscure, with any synergetic effects uncertain. This makes it unclear how to control and optimize the production of the desired products.

A peculiar characteristic of the PcCu 2D MOF is that it is obtained easily at the water–air interface, leading to a semiconductor with a low band gap of the order of 0.2–1.0 eV that is easy to exfoliate to obtain a monolayer or a few layers,^26^ which can be supported on a cathode for EC measurements. Recently, the 2D MOF in Figure 1 was shown to perform CO_2_RR with a FE = 50% (at −1.2 V_RHE_) for ethylene with additional products of methane and H_2_, each with ∼25% FE.^22^ A peculiarity of this particular MOF is that the catalytic cycle for C2 production involves only a single metal-atom (Cu) catalyst in the phthalocyanine core. This contrasts with the CO_2_RR for solution-based CuPc, which leads only to CO and the mechanism for CO_2_RR on Cu metal that involves 6 Cu atoms in the CO–CO dimerization step responsible for forming the new C–C bond. For a comparison of different 2D MOF in terms of FE and production rates, we refer to refs (22,27,28) and Table S7 in the Supporting Information.

In this paper, we report quantum mechanics-based computational studies on the PcCu-based 2D MOF to determine the reaction mechanism for forming methane and ethylene. We find a novel mechanism for carbon coupling to form ethylene that does not involve CO–CO dimerization.

## 2 Results and Discussion

### 2.1 Electronic Properties

We consider the 2D MOF in Figure 1a synthesized for the first time by Feng.^26^ This MOF was synthesized by linking the PcCu-(OH)_8_ macrocycle [(2,3,9,10,16,17,23,24-octahydroxyphthalo-cyaninato)copper(II)] with square-planar CuO_4_ nodes to create a 2D periodic structure (labeled PcCu). We used QM to optimize the MOF geometry and cell parameters (see the Methods section Supporting Information for more details), including potassium counterions, to balance the total charge of the system, making it as close as possible to the experimental conditions used for the CO_2_RR.^22^

From the total and projected density of states over the atoms (density of states (DOS), PDOS) analyses (Figure 1b), we observe that the main contribution to the valence band maximum arises from the carbon and oxygen linker atoms, while the conduction band minimum has additional contributions from the copper ions. Our density functional theory (DFT) calculations lead to a semiconductor with a narrow band gap of ∼0.3 eV, in agreement with experimental results and previous QM calculations.^26^ Examining the copper PDOS contributions, we observe that for the pristine MOF, the d_*xz*_ and d_*yz*_ orbitals contribute to the conduction band, while both d_*xy*_ and d_*x*^2^–y^2^_ orbitals contribute to the valence band, so that all Cu atoms have one unpaired electron, leading to a formal Cu^2+^ state with a d^9^ configuration (Figure 1c). Since the unit cell has three Cu atoms (one in the PcCu and two for each CuO_4_ ligand), the total system can be described as a *S* = 3/2 state.

Experimental evidence indicates that this PcCu MOF performs the CO_2_RR to obtain C2 products with high FE, but the reaction mechanism underlying this transformation remains unknown. CO_2_RR in this system is kinetically slow due to the extremely stable CO_2_, and the formation of different C1 and C2 products is very complicated since it proceeds through several proton-coupled electron transfer steps, ranging from

- 2e^–^ (HCOOH/CO),
- 4e^–^ (HCHO),
- 6e^–^ (CH_3_OH),
- 8e^–^ (CH_4_), up to
- 12e^–^ (C_2_H_5_OH/C_2_H_4_),
- and more for more complex products.^29−31^

Moreover, the reaction rate and yield of the target products are extremely low because of the sluggish kinetics and low solubility of CO_2_ in the electrolyte. In addition, at similar potential ranges, the HER process becomes more competitive, leading to lower CO2RR selectivity to CH_4_ and C_2_H_4_.

**Scheme 1:**
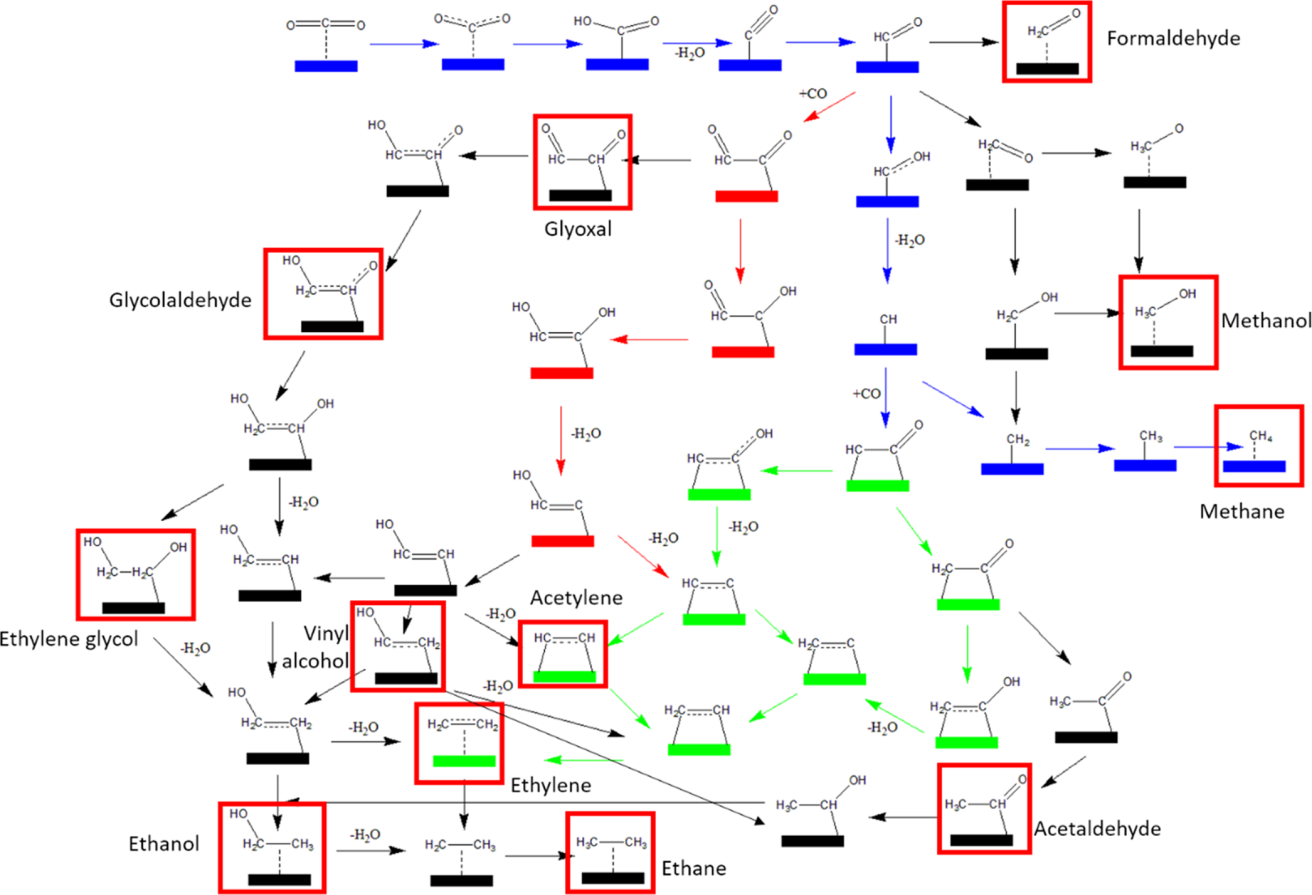
Full Reaction Pathway for CO_2_ Reduction to C1 and C2 Products In this paper, we focus on the formation of methane (blue path) and ethylene (green and red paths). The various possible intermediates that are most likely to be observed experimentally are labeled and indicated with red boxes.

We report here a series of QM calculations to discover the mechanism for the formation of C1 products (such as methane and methanol) and the formation of C2 products (such as ethylene and ethanol) (Scheme 1). In addition, we examine the possibility of forming other C2 products of industrial interest, including ethane, acetylene, acetaldehyde, vinyl alcohol, glyoxal, glycolaldehyde, and ethylene glycol, which are accessible via the shared formation pathway but have not yet been reported experimentally. Considering the complexity of these C2 formation paths, we limit the current study to the formation of methane (blue path in Scheme 1) and ethylene (green and red paths), while the other possible products will be the focus of subsequent studies.

### 2.2 Methane Formation Pathway

First, we consider the pathway for the formation of methane from CO_2_ adsorbed on the PcCu MOF. [The energies quoted in the paper are free energies in eV at 298 K, including zero-point energy, entropy, and the temperature contribution to enthalpy.] After favorable absorption with Δ*G* = −0.12 eV, the *CO_2_ is activated by bending, becoming stabilized by Δ*G* = −0.28 eV after overcoming an activation energy barrier of 0.32 eV. The first proton and electron transfer, with a barrier of 0.75 eV, leads to the formation of *HOCO, which is less stable than the *CO_2_ precursor by 0.15 eV. This difference in stability should be easily overcome at room temperature, with protonation of the OH for the first water elimination, leading to a barrierless formation of *CO. This *CO intermediate can be very stable, which might block subsequent catalytic processes.^32^ Indeed, we predict the overall stability of *CO to be Δ*G* = −2.27 eV compared to that of free CO_2_, with a binding energy of Δ*G* = −0.11 eV, suggesting that the catalytic process should proceed without problems. The small amount of CO observed experimentally (FE < 5% at −1.2 V_RHE_, which is the optimum potential for producing ethylene) can be the product of absorption over the different CuO_4_ moieties, with adsorption energy ranging from −0.18 to −0.20 eV. Moreover, this surplus of adsorbed CO molecules over the CuO_4_ moieties can be the source of molecules for the dimerization process to obtain C2 products. The *CO formed at the catalytic site can react with water to form *CHO (and OH) after an activation barrier of 0.22 eV. *CHO is the key intermediate for several pathways. From this point, we follow four distinct pathways:

(i) protonation of the carbonyl oxygen of *CHO to form *CH, eventually forming methane;
(ii) CO coupling to form *(CHO)CO to eventually produce ethylene and ethanol;
(iii) protonating the C to form *H_2_CO and eventually formaldehyde; or
(iv) protonating the same C toward production of methanol.

Interestingly, we did not observe the formation of the *COH intermediate, which on Cu metal surfaces is reported to lead to a competitive pathway with *CHO to form methane.^33^ We emphasize here that the *COH formation is strongly dependent on which facet of the Cu surface is considered, as *COH has been observed on the (100) facet, while on the (111) one, the pathway follows preferentially the *CHO formation.^33−35^

We focus here on the first two on pathways (i) and (ii), with the other two to be the focus of a future study. This is because the experiments have not yet searched for products in the aqueous solution such as methanol and ethanol.

**Figure 2:**
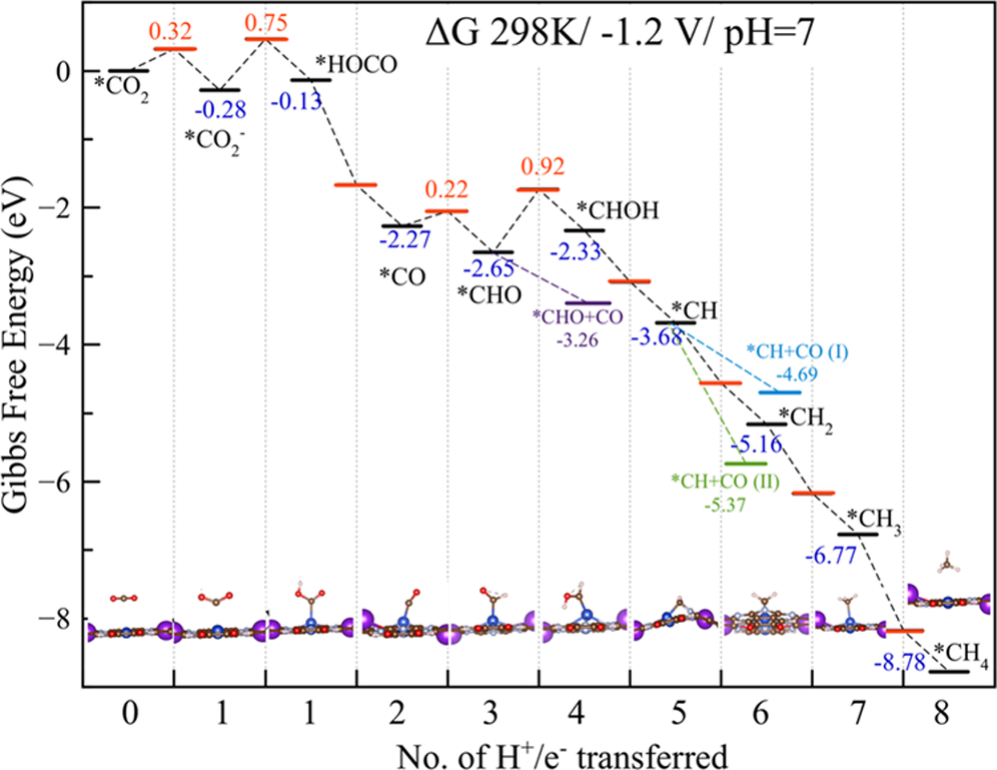
Energy diagram for the methane formation pathway (in black), computed at 298 K, neutral pH, at an applied potential of −1.2 V_RHE_. TS states and the relative activation energy barriers are indicated in red. Pathways to C2 products are indicated in purple, cyan, and green. Representative structures of the intermediates for methane formation are included.

Following pathway (i), the next step is protonation of the carbonyl oxygen to obtain *CHOH, which is less stable than *CHO by Δ*G* = 0.35 eV, making this the rate-determining step of the entire pathway with an overall activation energy of 0.92 eV (Figure 2).

On the other hand, coupling of CO leads to the barrierless formation of *(CHO)CO, which is more stable than the *CHO precursor by Δ*G* = −0.93 eV (as discussed in the next section). Thus, after the barrier of 0.92 eV to obtain *CHOH is overcome, this pathway may lead to a thermodynamically and kinetically stable pathway for the remaining steps toward the formation of methane. From this *CHOH intermediate, direct protonation of the carbonyl carbon will lead to *CH_2_OH, but we found this intermediate to be unstable, reverting back to *CHOH.

Instead, elimination of a water molecule by protonating *CHOH leads to the formation of *CH, which is stabilized by Δ*G* = −1.35 eV. From this *CH intermediate until the formation of methane, the reaction proceeds with no additional activation barriers. This strong stability of *CH arises from its peculiar structure, since it activates one of the nitrogen atoms of the Pc while distorting the planarity of the MOF. The strong interaction of CH with both Cu and N atoms is evidenced by the short bond distances C–N = 1.39 Å and C–Cu = 1.94 Å. We observe this peculiar geometry even when a second MOF layer is added to the MOF, suggesting that each layer is of sufficient structural flexibility to allow for this deformation. Formation of *CH leads to methane formation, even for a multilayer MOF. The subsequent *CH_2_ and *CH_3_ intermediates, obtained with coupled proton-coupled electron transfer (PCET) from solvent water, are more stable than their precursors by Δ*G* = −1.48 and Δ*G* = −1.61 eV, respectively. Interestingly, the distorted MOF framework remains after forming *CH_2_ but disappears for *CH_3_, confirming the flexibility of the 2D MOF framework. The final PCET leads to the formation of methane with Δ*G* = −8.78 eV compared to gas-phase CO_2_, but its binding energy is only Δ*G* = 0.01 eV, so that it desorbs readily from the surface at 298 K.

### 2.3 Ethylene Formation Pathways

One important consequence of the peculiar *CH structure is that it allows coupling of CO to obtain the (CH)CO intermediate that leads to ethylene production. This is because the *CH carbon atom is quite reactive (because of an incomplete electron shell). This provides a novel pathway for C2 product formation that does not involve the CO–CO dimerization commonly observed for electrocatalysis on Cu metal.^36,37^ After CO coupling into *CH to form the *(CH)CO intermediate, it is both kinetically (no activation barrier) and thermodynamically favorable, providing a pathway to ethylene that competes with methane formation.

We find two different pathways for the formation of ethylene.

**Figure 3:**
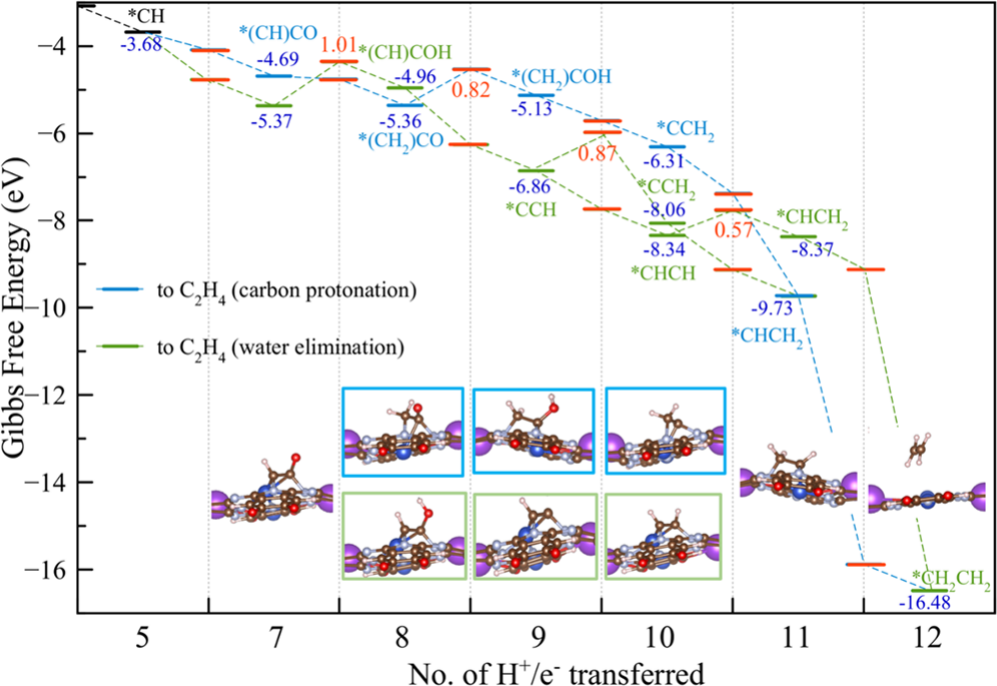
Energy diagram for the ethylene formation pathway from *(CH)CO, computed at 298 K, neutral pH, and an applied potential of −1.2 V_RHE_. Transition states and associated activation energy barriers are reported in red. The water elimination path is indicated in green, and the protonation path is indicated in cyan. Structures of the intermediates for ethylene formation are included.

**The first path** involves coupling of CO into the *CH intermediate to form *(CH)CO. This intermediate possesses a peculiar structure since the CH and the CO are each bonded to both Cu and a nitrogen atom of the Pc core. Indeed, activation of the first nitrogen atom for *CH formation is repeated with a second nitrogen atom when the CO is inserted, leading to *(CH)CO strongly interacting with both copper and nitrogen atoms. This involves short bond distances of C–N = 1.44 Å for CH and CO = 1.49 Å, with a similar Cu–C = 1.90 Å distance. This formation of *(CH)CO provides **two additional pathways** for subsequent steps:

(i) water elimination to form *CCH (depicted in green in Figure 3) or
(ii) carbon protonation to form *(CH_2_)CO (depicted in cyan in Figure 3).

This bifurcation into (i) and (ii) leads to two *(CH)CO intermediates with relative energies of Δ*G* = −4.69 and −5.37 eV, respectively, and no reaction barrier.

Following the water elimination mechanism, protonation of the carbonyl oxygen leads to the formation of the *(CH)COH intermediate with an energy of Δ*G* = −4.96 eV, which is 0.41 eV less stable than that of the *(CH)CO intermediate (at −5.37 eV), making this path less favorable for the formation of ethylene. This is further confirmed by the high activation energy barrier of 1.01 eV that must be overcome to obtain the *(CH)COH intermediate. Yet, if the temperature is sufficient to overcome this barrier (the rate-determining step for this pathway), one water molecule can be eliminated to form the *CCH intermediate, which is strongly stabilized at −6.86 eV, with no barrier.

From *CCH, there are **two different pathways** depending on which carbon atom is protonated, forming either *CCH_2_ or *CHCH. The formation of *CCH_2_ is kinetically unfavorable due to the activation barrier of 0.87 eV, whereas the formation of *CHCH is both kinetically favorable (no activation barrier) and thermodynamically stabilized by −1.48 eV, making it more probable. But *CHCH is only weakly bound to the copper atom (binding energy of only −0.02 eV), so it would probably lead to an acetylene product, which has not yet been observed experimentally.

Following the most probable path, the next coupled proton–electron transfer to *CHCH leads to the formation of the *CHCH_2_ intermediate, with virtually the same energy as *CHCH, being more stable by only −0.03 eV. However, to obtain *CHCH_2_, an activation barrier of 0.57 eV must be overcome. The final protonation of *CHCH_2_ leads to ethylene *CH_2_CH_2_, which is strongly stabilized by −6.75 eV. Since *CH_2_CH_2_ is weakly bonded to the surface with a binding energy of −0.17 eV, it should desorb readily.

Following the carbon protonation pathway at the CH moiety of *(CH)CO to form *(CH_2_)CO at −5.36 eV leads to a stable intermediate by −0.67 eV, with no activation barrier. From here, the carbonyl oxygen is hydrogenated to obtain *(CH_2_)COH, which is less stable than its precursor by 0.22 eV and additionally must overcome an activation barrier of 0.82 eV. Despite the small additional energy, forming this species becomes the rate-determining step of this whole path, which would control the rate for the formation of ethylene. Yet, if the temperature is sufficiently high, all of the following steps are barrierless. The next protonation to eliminate a water molecule leads to the formation of the *CCH_2_ intermediate, which is stabilized by −1.18 eV. From here, two additional coupled proton electron transfer steps lead first to the thermodynamically stable *CHCH_2_ and then to *CH_2_CH_2_, which is bound with respect to gas-phase ethylene by only −0.17 eV. Note that all intermediates involved in both pathways have a similar distorted MOF structure, which leads to a hollow shape (see Figure 3) to stabilize the whole system.

For this pathway, we predict that coupling of CO into *CH is not the preferable path because at least one transition state from each path requires a high energy barrier to be overcome, leading to intermediates that are less stable than its precursor. This happens for both pathways at the point at which the carbonyl oxygen is protonated to form either *(CH)COH or *(CH_2_)COH, leading to rate-determining steps of 1.01 and 0.82 eV, respectively. Since the first value is higher than the rate-determining step (of 0.92 eV) for the methane formation, we conclude that this water elimination path is not favored for the production of ethylene. Consequently, this explains why acetylene is not observed experimentally (it is formed only when the water elimination path is followed). On the other hand, the carbon protonation pathway can lead to ethylene formation since its rate-determining step (RDS) is lower than the RDS for methane formation by 0.1 eV. However, it must compete with methane formation, for which all intermediates obtained after *CHOH are sequentially more stable thermodynamically and kinetically favorable, due to the lack of energy barriers. Hence, the CO coupling into *CH might not contribute much to ethylene formation.

**Figure 4:**
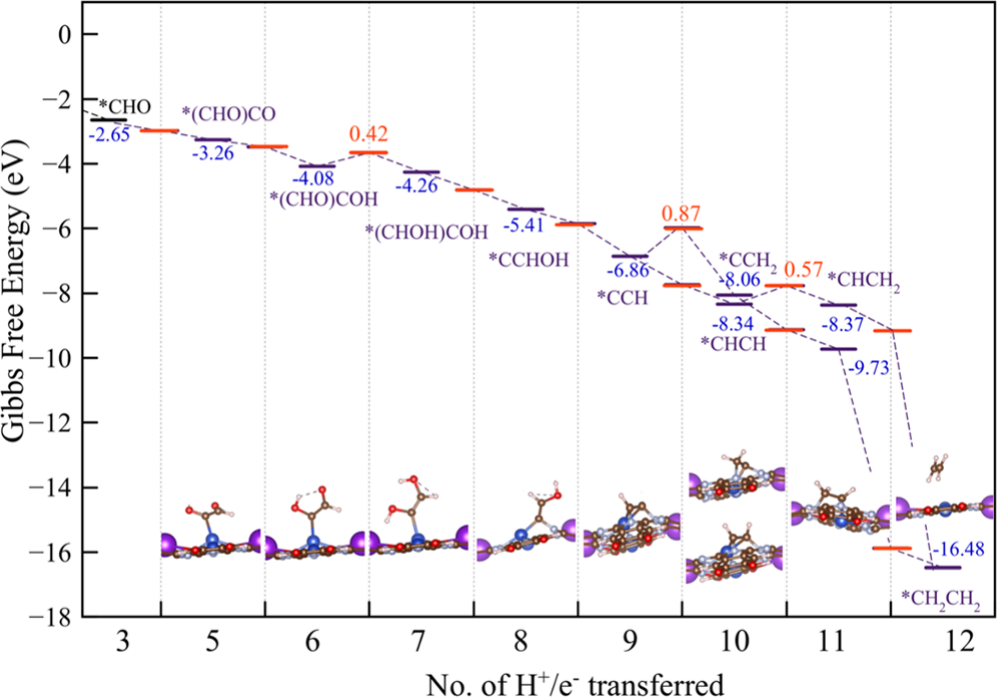
Energy diagram for the ethylene formation pathway from *(CHO)CO, computed at 298 K, neutral pH, and an applied potential of −1.2 V_RHE_. The transition states and the associated activation energy barriers are reported in red. Intermediate structures for ethylene formation are included.

**The second pathway** arises from the coupling of CO into the *CHO intermediate (Figure 4). This formation of *(CHO)CO leads to an additional stability of Δ*G* = −0.93 eV with respect to the parent intermediate, and can be formed without an activation energy barrier. From here, there is a favorable pathway in which each intermediate is more stable than its precursor, with only one activation energy to be overcome. In particular, after CO coupling, we find two consecutive protonations of carbonyl oxygen atoms, both leading to stable intermediates. The first one, *(CHO)COH, is strongly stabilized by Δ*G* = −0.81 eV, while the second, *(CHOH)COH, is stabilized by only Δ*G* = −0.18 eV and requires overcoming an energy barrier of 0.42 eV, making formation of this intermediate the rate-determining step for this pathway. Nevertheless, this energy barrier is much smaller than the 0.92 eV obtained for the formation of *CHOH leading to methane, thus making this pathway more favorable. Next, the first water elimination leads to the formation of *CCHOH, which is stabilized by Δ*G* = −1.15 eV. This leads to a bent MOF structure (as observed for *CH). In fact, the carbon atom closer to PcCu loses its OH group, making it strongly reactive since one bond is missing to complete the carbon valence shell. To stabilize its structure, this carbon atom interacts with a nitrogen of the Pc core, activating it to complete its valence while making the nitrogen pyramidal. In this configuration, the nitrogen forms a bond with the carbon atom (C–N = 1.44 Å) to stabilize the structure. Then, elimination of a second water molecule from *CCHOH leads to the formation of the *CCH intermediate, in which the second carbon atom has an incomplete valence. To stabilize this structure, it forms a bond with the copper atom (Cu–C = 1.91 Å).

From the *CCH intermediate, protonation can occur either on the carbon atom already protonated, leading to *CCH_2_, or at the second carbon atom, leading to acetylene *CHCH. But acetylene formation is favored both kinetically and thermodynamically, with its formation energy stabilized by Δ*G* = −1.48 eV in contrast to *CCH_2_, which is destabilized by Δ*G* = 0.27 eV while requiring an activation energy barrier of 0.87 eV. This suggests that formation of acetylene might be experimentally observable (it has not been reported), although it might be with a small yield.

From *CHCH, an additional protonation leads to *CHCH_2_ and then to ethylene.

Overall, the coupling of CO into the *CHO intermediate seems to be the most probable path for formation of ethylene, since all intermediates are more thermodynamically stable than their precursors, and the rate-determining step has lower energy than the pathway leading to methane. This pathway might also lead to the formation of acetylene. Considering the entire pathway from CO_2_ to ethylene, we predict that ethylene formation is strongly favorable, rationalizing the experimentally observed very high faradic efficiency of 50%.

As the GCP-K method is used here for the first time on a semiconductor substrate for electrocatalysis, it is worth comparing these results with the more commonly used computational hydrogen electrode (CHE) method in which the charges are kept constant. As reported in Figures S6–S8, there is a big discrepancy in the stability of the intermediates between the two methods. With the CHE, one strongly overestimates the thermodynamic stability of all of the intermediates. Indeed, in some cases, CHE wrongly describes activation energies. The CHE method can still be useful for the C1 product at a qualitative level since the discrepancies are not so severe. However, CHE fails for more complex reactions, where different pathways can be present simultaneously. Thus, for C2 products, CHE gives incorrect energetics for both the intermediates and for the activation energies, leading to possible incorrectly favored reaction pathways.

### 2.4 Optimizing the Ethylene Production Yield

Examining closely the energy diagram in Figure 2, we observe that a key step is the competition between formation of *CHOH and *(CHO)CO from *CHO. As mentioned above, *CHO is the key intermediate not only for both methane and ethylene production but also (among others) for methanol and ethanol production. To understand and rationalize the mechanism and also the efficiency of the entire catalytic cycle, we focus next on the relative stability of the intermediates and transition states.

**Figure 5:**
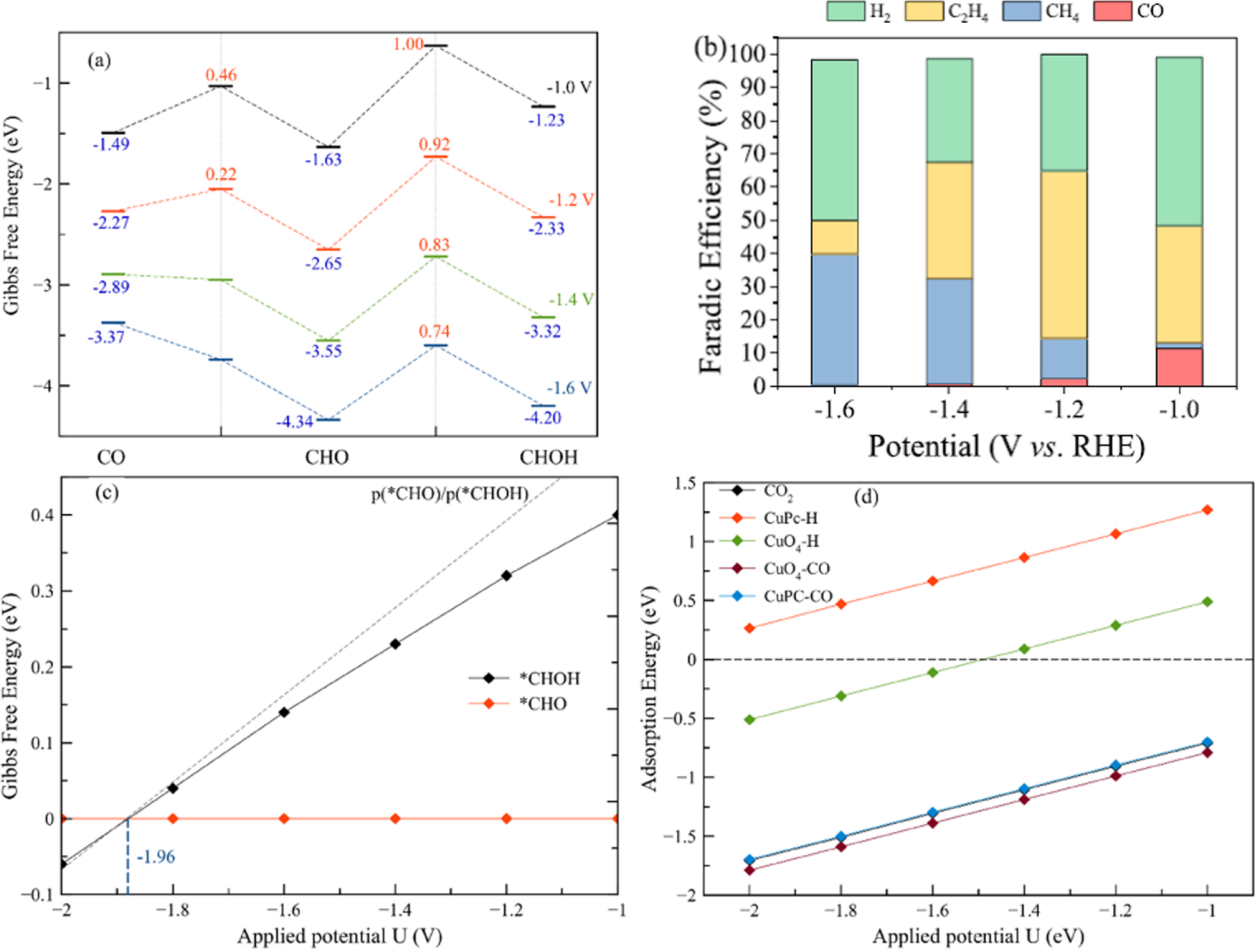
(a) Energetics of three selected intermediates and transition states that are key to the formation of both methane and ethylene as a function of applied potentials. (b) Experimental faradic efficiency at various applied potentials [adapted with permission from ref (22). Copyright 2021 American Chemical Society]. (c) Relative Gibbs free energy of *CHO and *CHOH at various applied potentials, with reference to *CHO. The calculated Boltzmann factor between these two configurations is shown as a gray dotted line. (d) Adsorption energy of CO_2_, H, and CO at various applied potentials and at different adsorption sites.

From our discussion above, it is clear that the most probable path for the formation of ethylene is CO coupling into *CHO, since all reaction steps are exoergic and barrierless. Moreover, from Figure 2, it is clear that the rate-determining step distinguishing the methane and ethylene pathways is the formation of *CHOH from *CHO. Thus, if the energy difference between these two intermediates and the relative transition state is high and favors *CHO, production of ethylene is increased while methane production is decreased. In contrast, decreasing the difference in energy between these intermediates, as well as the barrier between them, favors methane. Indeed, this is observed experimentally as the applied potentials are changed (Figure 5b): at a low applied potential of −1.0 V, formation of methane is suppressed, but changing the potential to −1.6 V strongly increases methane production while ethylene production almost stops.

To compare our calculations with experiments, we applied the grand canonical potential kinetics (GCP-K) method (also known as grand canonical QM or GC-QM) to predict the free energy of three selected intermediates (*CO, *CHO, and *CHOH, Figure 5a) and their transition states as a function of applied potential over the same range as the experiments. As shown in Figure 5a, we predict that *CHO formation becomes barrierless at −1.4 V, with the stability of the intermediate steadily increasing with increasing applied potential. Moreover, at a low potential of −1.0 V_RHE_, the energy of *CHOH is as much as 0.40 eV higher than that of *CHO, leading to high ethylene production and negligible methane production (as observed experimentally). This low production of CH_4_ is also due to the high energy barrier of 1.00 eV that must be overcome in this step. Changing the potential to more negative values leads to overall stabilization of all selected intermediates and the transition states. However, while the energy difference between *CHO and *CHOH steadily and strongly decreases, going from 0.40 eV at *U* = −1.0 V to 0.14 eV at *U* = −1.6 V, the decrease in the energy barrier is rather small, from 1.00 at *U* = −1.0 V to 0.74 eV at *U* = −1.6 V. The small difference in energy predicted at −1.6 V confirms that these are the key intermediates to consider in shifting the production toward either methane or ethylene. We predict that a potential of −1.2 V leads to the maximum yield of ethylene while decreasing methane formation, due to the difference in energy barriers to overcome in the rate-determining steps of the two pathways of 0.92 eV for methane and 0.57 eV for ethylene. At the same time, changing the applied potential toward more negative values makes the methane pathway more favorable, since now the rate-determining step for *CHOH formation is lower (0.74 eV at −1.6 V) and can become more competitive with the ethylene formation path. At −1.2 V, the difference in energy between *CHO and *CHOH is 0.92 eV, which ensures a high production of ethylene. Even if this energy barrier is overcome and the path to methane becomes accessible, its formation might still be hindered by the CO coupling pathway into *CH, thus maximizing the ethylene production while minimizing methane formation.

This can also be understood by considering the Boltzmann probability distribution for the formation of *CHO and *CHOH (Figure 5c) as a function of applied potentials. The two lines cross at −1.96 V, indicating the potential above which the *CHOH population is expected to be higher than *CHO (assuming no kinetic and diffusion limitations). The probability of two states is calculated using the Boltzmann factor so that the ratio of probabilities of the two states i and j depends only on their energy difference
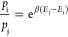
1This estimate (reported in Figure 5c as a gray line) indicates that after the applied potential is changed to −2.0 V, the *CHOH population is greater than that of *CHO by a factor of 100.

Yet, this increase of the external bias not only shifts the mechanism toward an enhanced production of methane, but it also increases the detrimental hydrogen evolution reaction (HER). On the present 2D MOF, there are three possible adsorption sites for hydrogen (as for any other gas-phase molecules present in the electrolyte such as CO_2_ water and CO), namely, at the phthalocyanine copper (CuPc) or at the two CuO_4_ moieties. The adsorption energy of H, CO, and CO_2_ at different applied potentials on the two different adsorption sites is reported in Figure 5d. Despite not being in the full reaction path, this adsorption energy analysis indicates how easy it is for a species to interact with the catalytic center, helping rationalize the different processes that can occur at the three different metal centers. When no external potential is applied, we find adsorption energies of −0.12/–0.11/1.85 eV for CO_2_, CO, and H, respectively, at the PcCu catalytic center. Thus, making the potential more negative makes the adsorption energy more favorable for all of these species. We emphasize that hydrogen has a positive adsorption energy (unfavorable) at all applied potentials when interacting with the CuPc catalytic center, while both CO and CO_2_ show strong interactions. This leads to two conclusions. First, the HER is likely to take place at the CuO_4_ moiety and not at the CuPc center responsible for the CO_2_RR. In fact, the H adsorption energy becomes more favorable at these CuO_4_ centers at more negative applied bias, explaining the enhanced experimental FE for H_2_ formation at potentials more negative than −1.4 V. Second, both CO and CO_2_ have favorable interaction with the CuPc catalytic center, strongly indicating that their reactions can proceed without loss of a key intermediate such as CO. Interestingly, the adsorption energy of CO on the CuO_4_ moiety is also favorable but weak, with similar values of −0.20 and −0.18 eV for the first and second adsorption at zero bias, respectively. The difference in adsorption between the two sites, reflecting a different catalytic activity, can also be quantified and justified by considering the d-band center density of states (DOS) of the different metal centers. In fact, the main idea is that the closer to the Fermi energy that the metal d-band center is, the stronger the catalytic activity is, due to a lower occupation of antibonding states. For the different catalytic sites, we obtain a d-band center value of −2.64 eV for CO–CuPc, −2.85 eV for CO–CuO_4_, and −2.92 eV for 2CO–CuO_4_ (Figure S9). Hence, we observe electron redistribution of the 2p orbitals of CO, which leads to a positive shift of the Cu 3d band toward the Fermi level for the CuPc center, which is accompanied by a decreasing antibonding occupation for the CuPc center. Taken together, this allows us to reason that CuPc has enhanced catalytic activity compared to the CuO_4_ centers, making it the main catalytic center for the CO_2_RR. This might also explain why the FE for ethylene strongly decreases at a potential more negative than −1.2 V. In fact, as the CO interaction becomes stronger, the reservoir of molecules needed to promote the ethylene pathway is not available due to the strong CO–CuO_4_ interaction. A second possible explanation is that at the potential of −1.6/–1.8 V, hydrogen adsorption becomes competitive, with the CuO_4_ centers becoming the catalytic centers for the HER process.

## 3 Experimental Section

All calculations were performed using spin-polarized density functional theory (DFT) as implemented in the Vienna Ab initio Simulation Package (VASP). We used VASPsol to include implicit solvation, with water solvent parameters.^38−42^ We used the Perdew–Burke–Emzerhof (PBE) functional with a plane-wave cutoff energy of 500 eV. For structural optimizations, the Brillouin zone was sampled using a 1 × 1 × 1 *k*-point grid based on the Monkhorst–Pack scheme. We used a vacuum space of 1.5 nm in the *z* direction (perpendicular to the MOF basal plane) to avoid interactions between the periodic images. The convergence criteria for the force on each atom was set to 0.02 eV/Å, while the electronic structure energy convergence criterion was 10^–5^ eV. We employed the Grimme D3 method with Becke–Johnson parameters^43^ to account for van der Waals interactions.^44^ The vibrational modes were calculated at 298.15 K to obtain the zero-point energy, entropy, and temperature corrections to enthalpy.

To accurately describe proton transfer from water solvent to the reactant, we considered the solvent as follows: a four-water-molecule cage was added to the system, in which one is protonated, and this cluster is inserted close to the reaction center to enable the proton transfer process. Moreover, the whole system was also surrounded by implicit solvent, giving an additional layer of solvation, which can affect the catalysis. The implicit solvent was considered to be within the VASPsol method. We optimized the geometry of the MOF structure with only the core of the phthalocyanine, the adsorbate, and the water cluster free to move while the rest of the system was kept frozen to retain the planarity of the MOF basal plane. After obtaining the optimized structures, we performed single-point JDFTx calculations at various applied potentials using the CANDLE implicit solvation model to provide a more accurate description of the solvent and to obtain the free energy.^45,46^ We then applied the Legendre transformation to obtain the grand canonical potential energy GCP(U).^47^

Since CO2RR is an electrochemical process, we use the recently developed grand canonical potential kinetics (GCP-K) so that the reactant, transition, and product states are all at the same applied potential, just as in experiment.^47−49^ Since the applied potential is constant, the charge near the active site changes as the reaction proceeds from the reactant state through the transition state to the product. In standard QM, keeping the chemical potential constant as the QM wave function structure is optimized along the reaction path is challenging and time-consuming. However, the GCP-K methodology greatly simplifies the procedure, making it very efficient. In GCP-K, the total free energy, *F*(*n*), is calculated for each reaction step using standard QM as a function of the net charge. Then, we apply a Legendre transformation to convert *F*(*n*) to the grand canonical free energy, G(*n*; *U*)
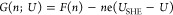
2where the grand canonical free energy *G* depends on the number of electrons (*n*) and the applied potential (*U* vs SHE), where *F*(*n*) is the total free energy as a function of the number of electrons and *U*_SHE_ = μ_e,SHE_/e is the electronic energy at standard hydrogen electrode (SHE) condition. Thus, we shift the Fermi level to the applied potential by changing the electronic band occupation and varying the number of electrons in the systems. For each potential (*U*), the number of electrons is optimized, dG(*n*; *U*)/d*n=* 0, leading to the grand canonical potential GCP(U), where the number of electrons depends implicitly on the potential *U*. Approximating *F*(*n*) locally as a quadratic function and minimizing *G*(*n*; *U*) leads to a quadratic form for GCP(*U*), which accounts for the change in capacitance as the potential changes.

The accuracy of the GCP-K method has been recently validated for several different electrochemical reactions taking place on different metal substrates, including Co/TiO_2_ single-crystal nanoparticles,^50^ Ni single sites in graphene,^48^ and semiconductor chalcogenides (WSe_2_ and MoTe_2_).^47,51−53^

Activation energies were computed using the Brønsted–Evans–Polanyi (BEP) relationship,^54,55^ which relates the kinetic barrier to the corresponding reaction energy for a class of materials, which has been recently validated for electrochemical reactions.^56^ The main approximation in this approach is that the transition state located for the nonelectrochemical step is equivalent to that of the electrochemical step at a specific potential *U*:
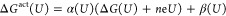
3where α(*U*) and β(*U*) represent the best fit slope and intercept of the BEP scaling relation that vary with potential. Since we use the GCP-K method, we already have the free energy at the fixed potential of −1.2 V. Thus, the β parameter becomes a potential-independent constant that is typically between 0.3 < β < 0.7, with the most often used value of 0.5, while α is set to 1. As a result, we can predict the potential-dependent activation energy (Δ*G*^act^ (*U*)) for an elementary reaction, given the value of Δ*G*(*U*), of *U*, and an approximate potential-independent β, without the need to conduct an expensive, time-consuming, and computationally demanding DFT barrier calculations.^57^

We took the input structure of the MOF cell from the literature^26^ and reoptimized it with the QM method, as described above. We neutralized the system by including 4 potassium cations that coordinate with the O atoms of the CuO_4_ moiety. To ensure the correct description of the different Cu_2_^+^ ions in the system (one on the phthalocyanine and two on the CuO_4_ linker), we consider spin polarization for the *S* = 3/2 high spin case during the geometry optimization. The final cell dimensions are *a* = 18.17536 Å, α = 91.3834°, *b* = 18.17632 Å, β = 91.0939°, *c* = 20.20189 Å, and γ = 89.8539°. From the optimized MOF monolayer, we computed the electronic properties reported in the Section 2.

## 4 Conclusions

In this paper, we present the results from QM computational studies for the reaction mechanism of CO_2_RR electrocatalysis of a novel 2D MOF, which acts as a single-atom catalyst to produce ethylene and methane. Using the grand canonical potential kinetics (GCP-K) QM method, we observed that at the working overpotential of −1.2 V, there is one thermodynamic favorable pathway to obtain methane, while there are two pathways for producing ethylene. The first ethylene pathway considers CO coupling into the highly reactive *CH intermediate, which activates the N atoms in the basal plane of the 2D MOF to form *(CH)CO. From here, we find two pathways driven by either water elimination or carbon protonation. While water elimination is not the favorable pathway due to the large activation barrier (>1.00 eV), carbon protonation is more favorable, since the barrier to overcome is lower than the rate-determining step for methane formation.

The second pathway for ethylene formation considers the coupling of CO into the *CHO intermediate to form *(CHO)CO. This path is both kinetically and thermodynamically favorable, leading to ethylene production with no high energy barriers to overcome. Moreover, we predict that the formation of acetylene is a possible secondary product. The key intermediate for C2 production is the *CHO intermediate, which leads to C–C dimerization in a thermodynamically favorable pathway toward ethylene. This is in contrast with the CO–CO dimerization step commonly observed for ethylene production on Cu metal surfaces. The competition between dimerization and protonation of *CHO is the key to controlling the yield for forming ethylene and methane, where fine-tuning of the applied potential can be used to optimize ethylene production.

We showed that the grand canonical potential computational method provides an extremely powerful tool both to demonstrate and unravel the reaction mechanism to address the mechanism underlying experimental results and also to make accurate predictions on the different possible products of CO_2_RR under different experimental conditions, such as pH, overpotential, and electrolyte composition.

With this validated tool at hand, we plan to expand the study to other possible products that might be dissolved in the aqueous medium and not yet reported (such as methanol and ethanol), to assess in detail the role of the different metal centers in propagating the reactions (in particular, to assess the source of CO for the dimerization process), to optimize the selective production of C2+ solar fuels by changing the catalytic metal center (from Cu to Fe, Co, Ni, Mn), and to modify the hardness/softness of the whole structure by modifying the other two metal centers. The aim of these different studies will be to obtain the best catalyst and catalytic conditions to ensure the highest possible faradic efficiency of the reaction toward the selective formation of the desired product while simultaneously suppressing the undesired HER process.

## Supporting Information Available

Including parameters for the GCP-K method, reaction pathways at acid pH, comparison between CHE and GCP-K performances, additional details on the derivation of the GCP-K, and additional electronic properties analyses (PDF)
