# Supplementary material for: CO2 Reduction to Methane and Ethylene on a Single-Atom Catalyst: A Grand Canonical Quantum Mechanics Study
Source: J Am Chem Soc. 2023 Sep 20;145(39):21319–29. doi: 10.1021/jacs.3c05650 (PMC10557142; doi:10.1021/jacs.3c05650)
Supplement: Supplementary file 1 — ja3c05650_si_001.pdf [file ja3c05650_si_001.pdf]

Supporting Information for

CO_2 reduction to methane and ethylene on a single atom catalyst: a grand canonical quantum mechanics study

Silvio Osella,[1,2] William A. Goddard III[2]

[1] Chemical and Biological Systems Simulation Lab, Centre of New Technologies, University of Warsaw, Banacha 2C, 02-097 Warsaw, Poland.

[2] Materials and Process Simulation Center (MSC), California Institute of Technology, MC 139-74, Pasadena CA, 91125, USA.

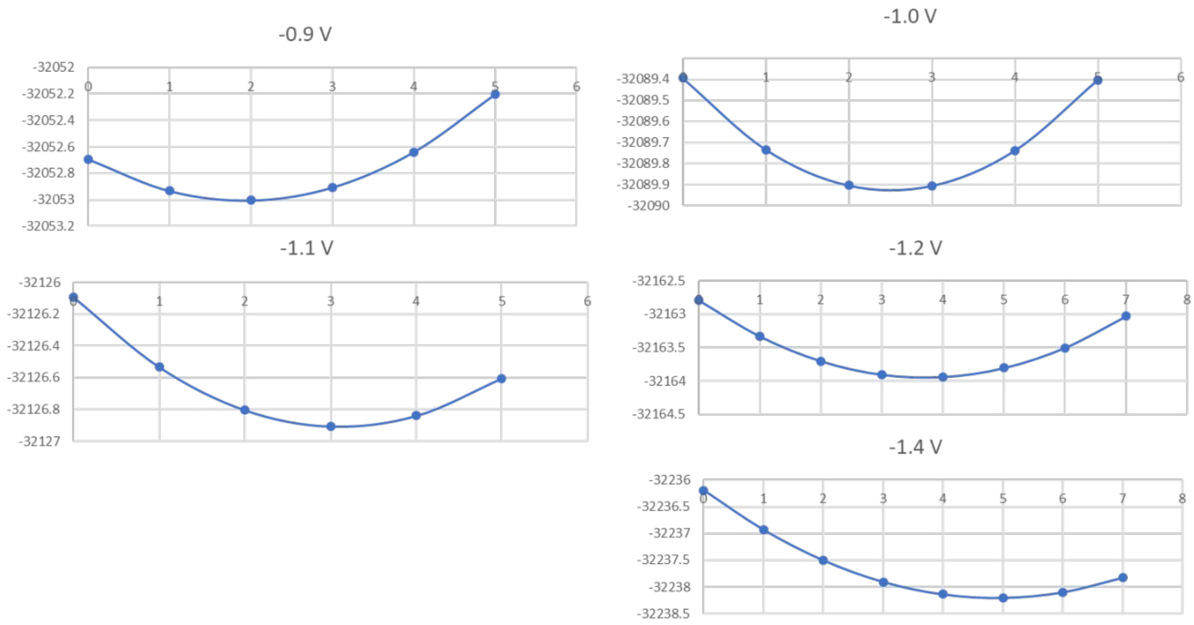

Fig. S1. Free energy vs. number of electrons profiles for different applied potentials for *CO adsorbed on CuPc, using the GCP-K method.

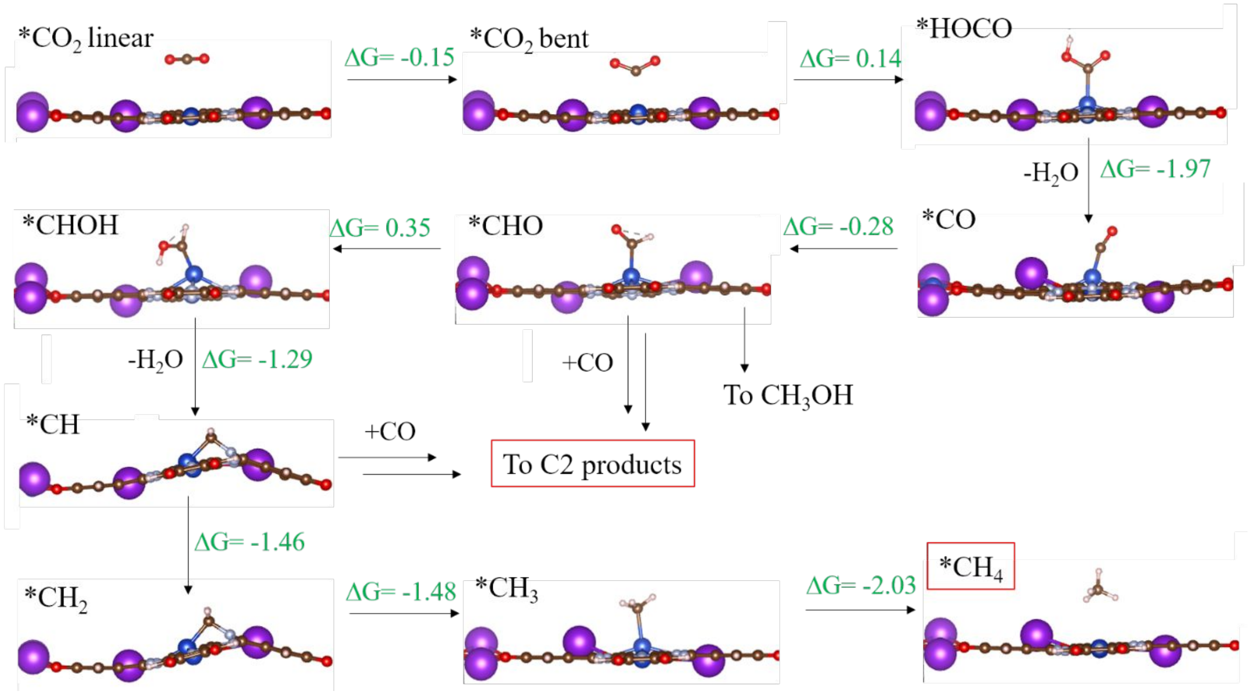

Fig S2. Representative structures of the intermediates for methane formation. Gibbs free energy changes are reported for U=-1.2 V and pH=5.67, at 298 K (experimental conditions). All energies are in eV.

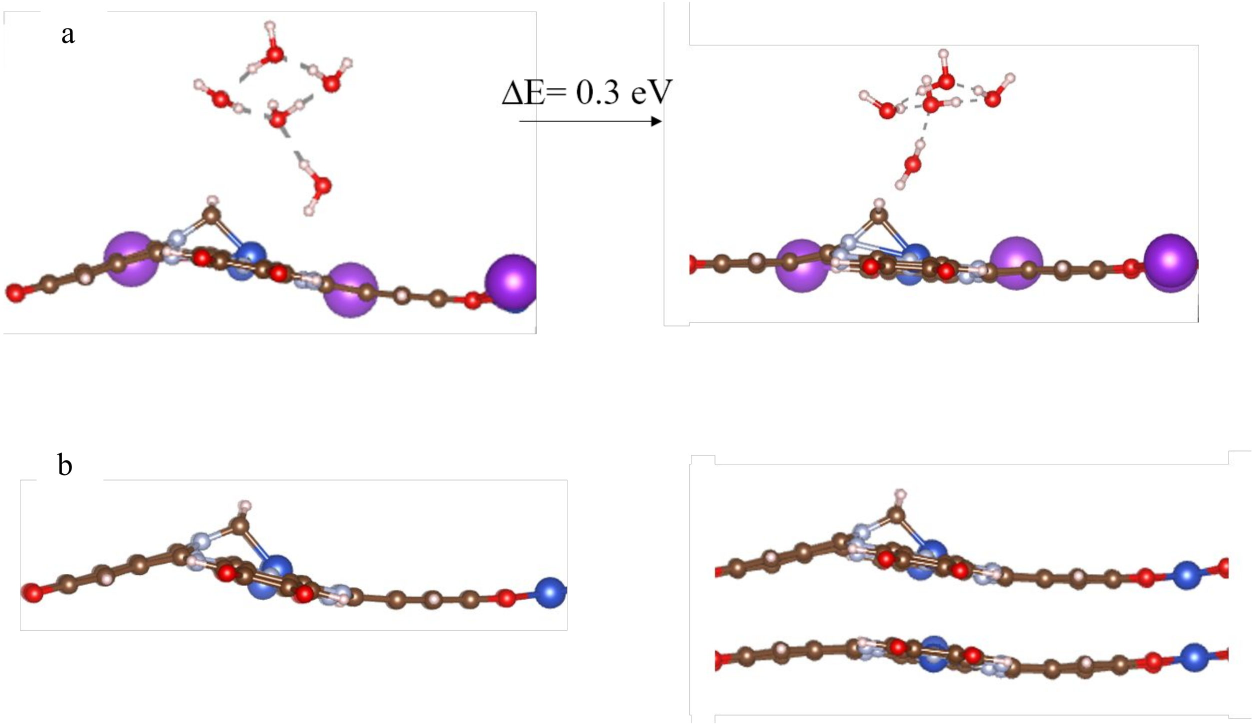

Fig S3. Conformation of the *CH intermediate, in which one nitrogen atom of the MOF is activated, leading to a bending of the basal plane of the MOF. a) Optimized monolayer without constrains in the structure (left) is more stable than the optimized structure with constrains on the MOF (right) of 0.3 eV. The water cluster used for all calculations is also shown. b) the activation of the nitrogen atom is present even when a second layer is added to the model system, confirming the flexibility of the 2D-MOF structure.

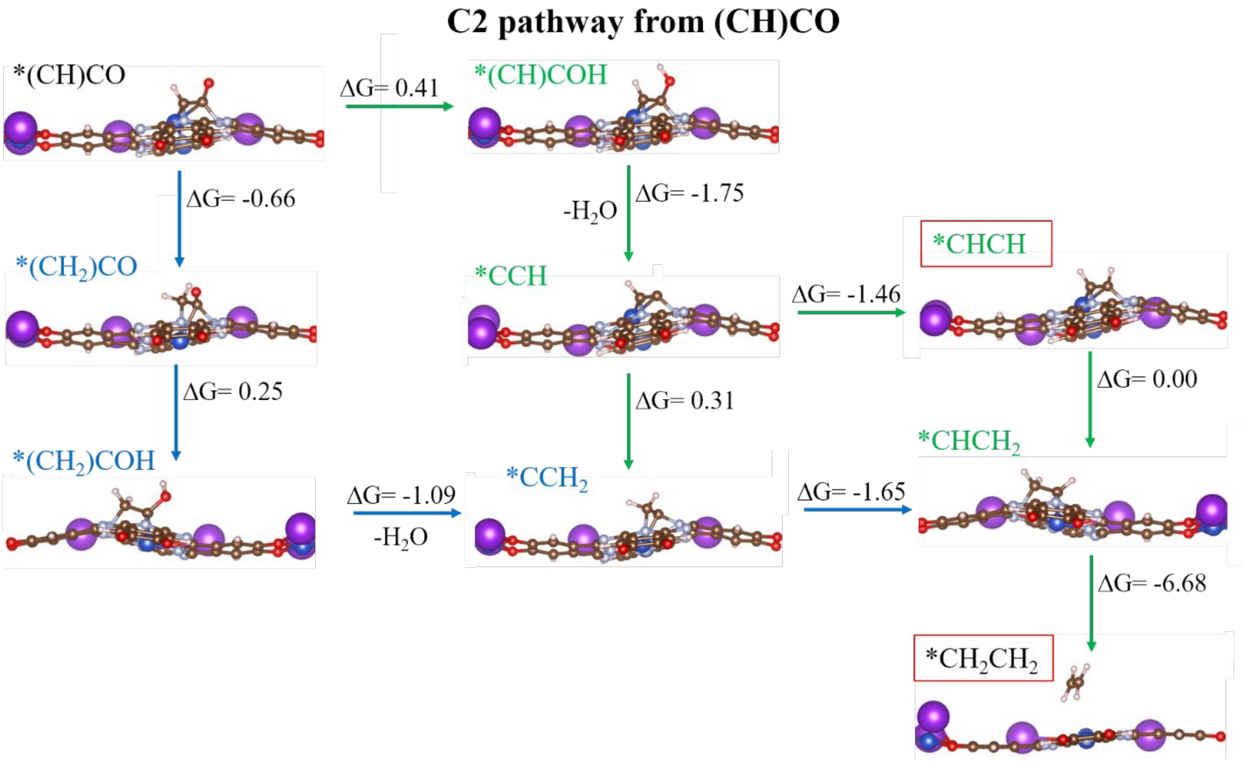

Fig S4. Representative structures of the intermediates for ethylene formation from *(CH)CO. Gibbs free energy changes are reported for U=-1.2 V and pH=5.67, at 298 K (experimental conditions). All energies are in eV.

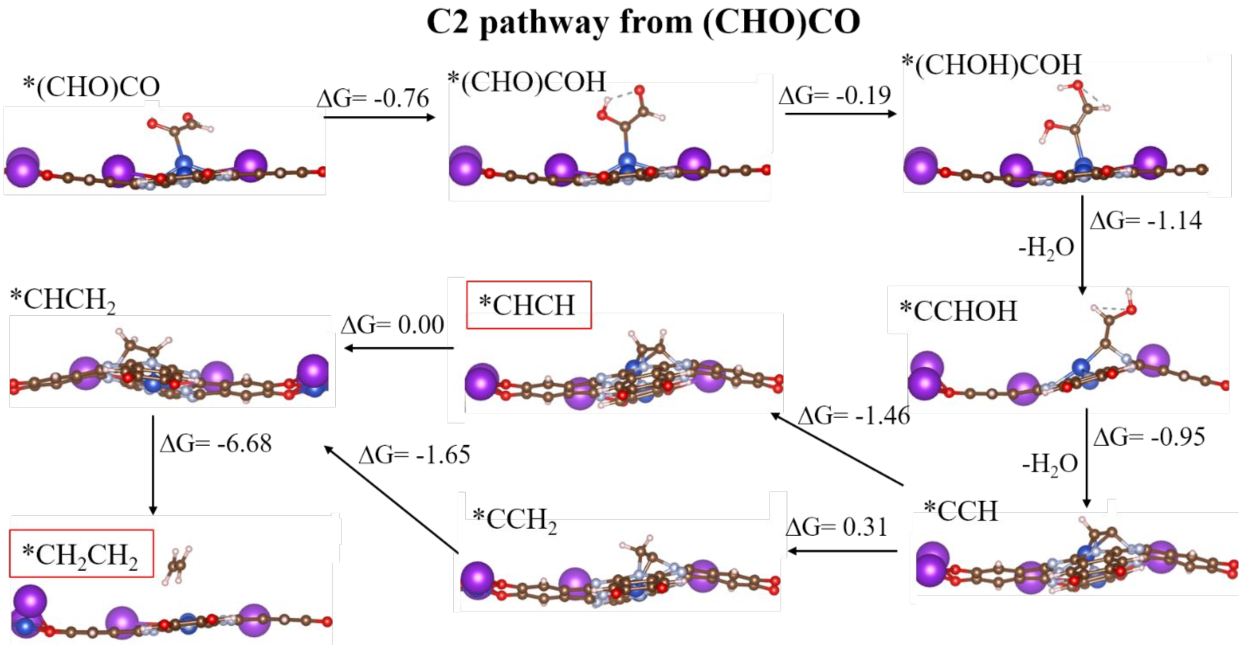

Fig S5. Representative structures of the intermediates for ethylene formation from *(CHO)CO. Gibbs free energy changes are reported for U=-1.2 V and pH=5.67, at 298 K (experimental conditions). All energies are in eV.

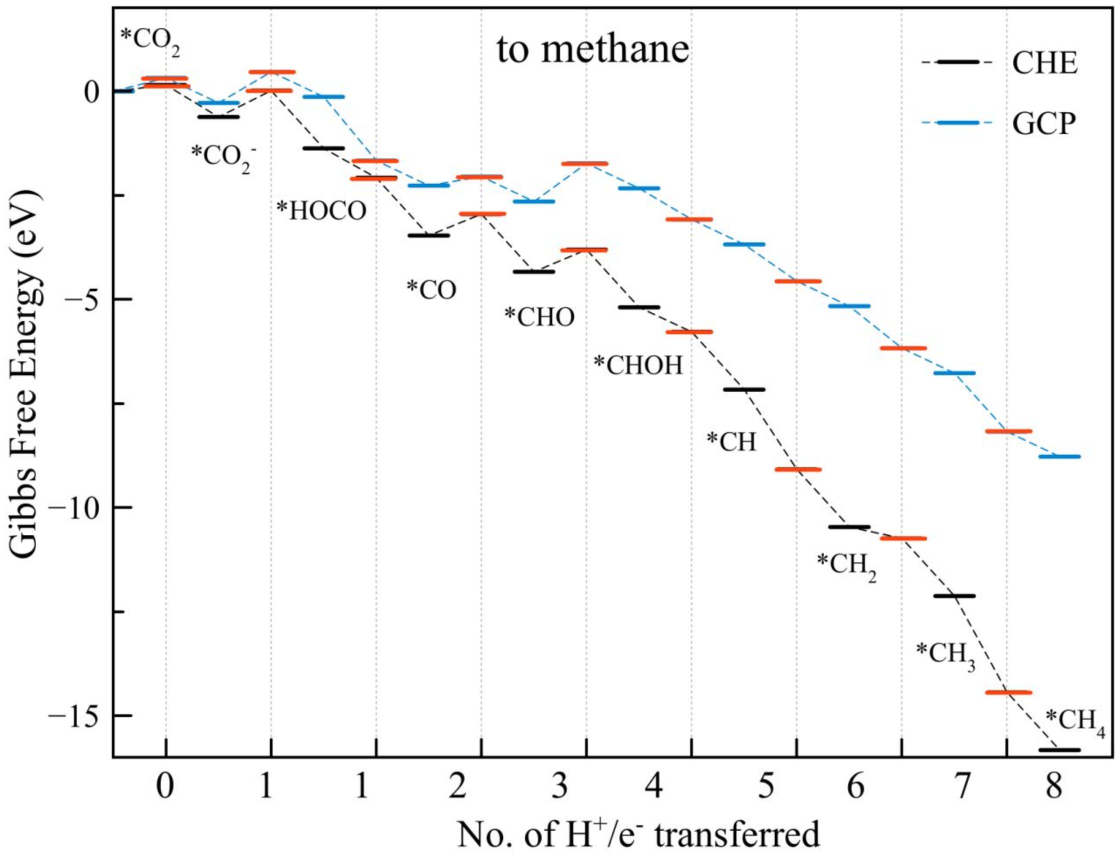

Fig S6. Energy diagram for methane formation using the CHE and the GCP models. Gibbs free energy changes are reported for U=-1.2 V and pH=7, at 298 K (experimental conditions). All energies are in eV. The transition states and the associate activation energy barriers are reported in red.

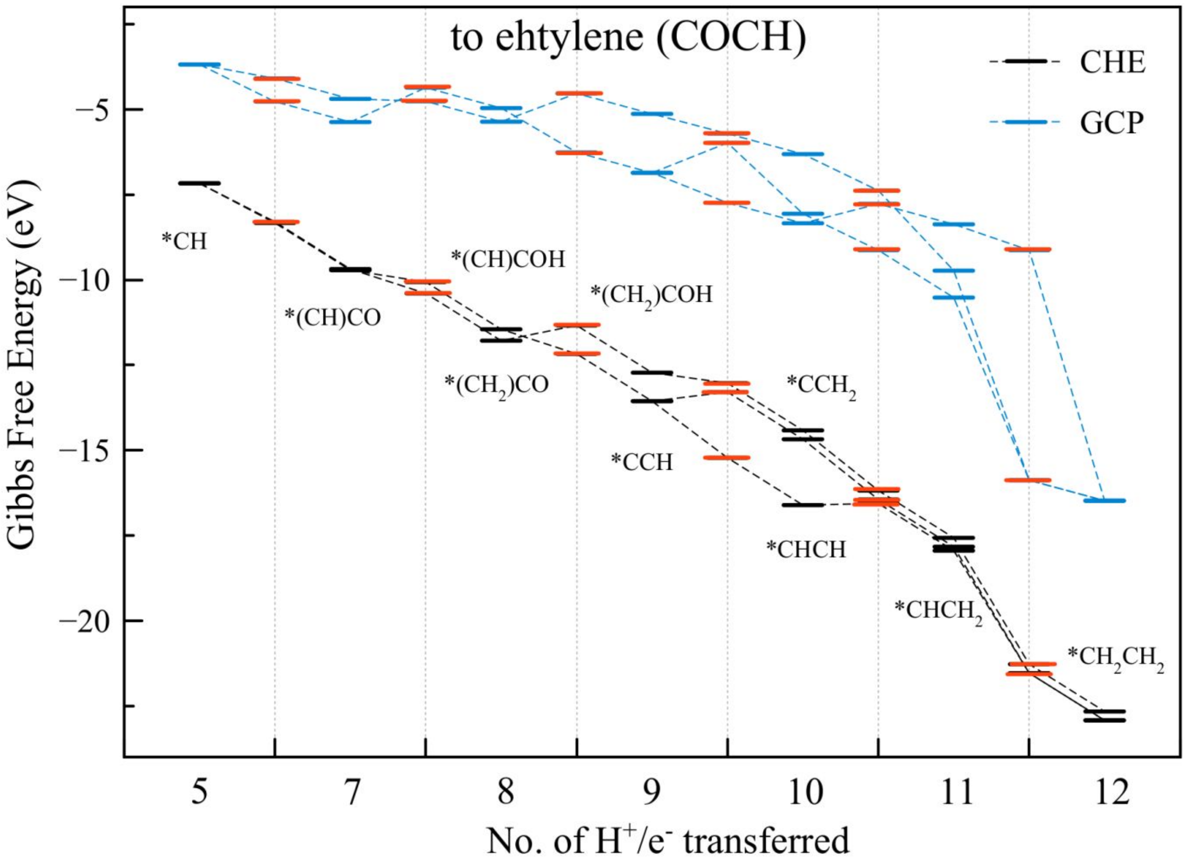

Fig S7. Energy diagram for ethylene formation along the *(CH)CO pathway using the CHE and the GCP models. Gibbs free energy changes are reported for U=-1.2 V and pH=7, at 298 K (experimental conditions). All energies are in eV. The transition states and the associate activation energy barriers are reported in red.

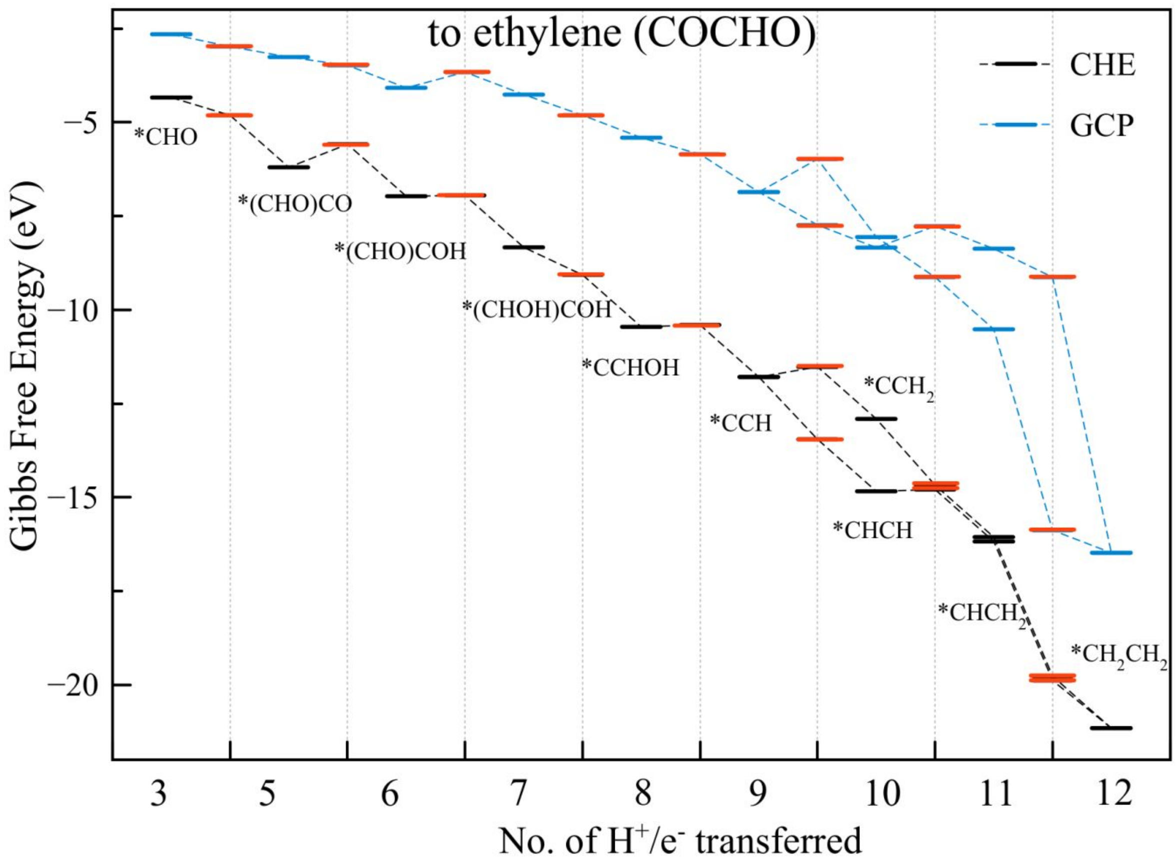

Fig S8. Energy diagram for ethylene formation along the *(CHO)CO pathway using the CHE and the GCP models. Gibbs free energy changes are reported for U=-1.2 V and pH=7, at 298 K (experimental conditions). All energies are in eV. The transition states and the associate activation energy barriers are reported in red.

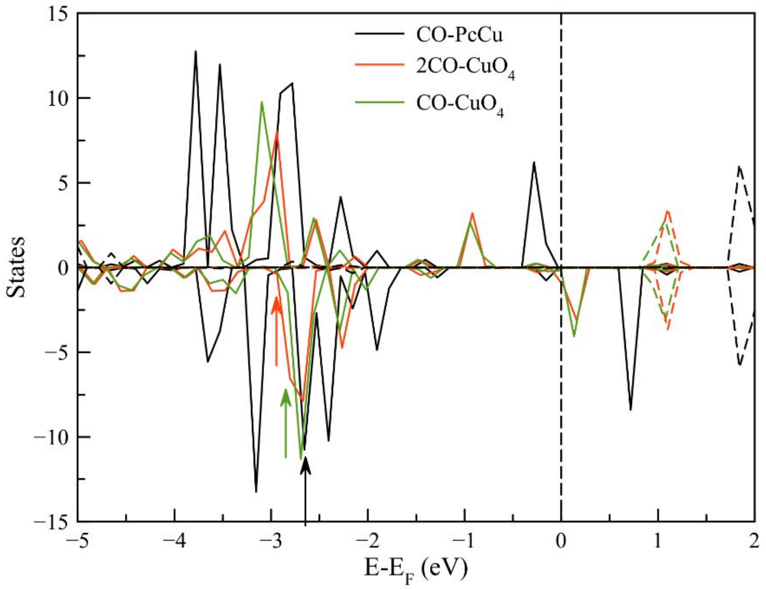

Fig S9. Partial density of states for the 3d orbitals of the different Cu sites (solid lines) and 2p orbitals for the CO adsorbate (dashed lines). The arrows indicate the d-band position for the different catalytic centers. The vertical dashed line refers to the Fermi Energy.

Table S1. Gibbs free energy vs. number of electrons profiles for the intermediates leading to CH_4, obtained using the GCP-K method, at -1.2 V, pH=7 and T=298 K. Energies are reported in eV.

*CO_2	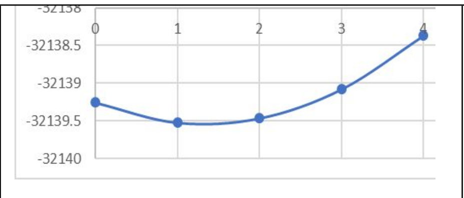	*CHOH	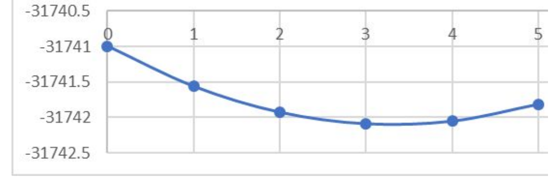
*CO_2 bent	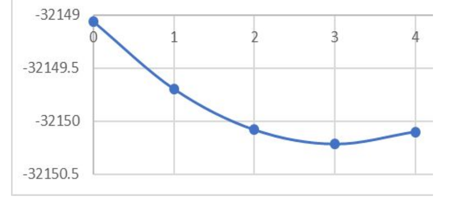	*CH	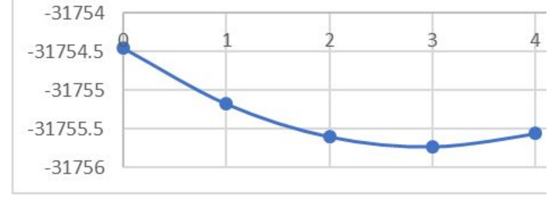
*HOCO	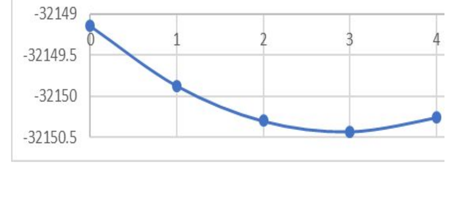	*CH_2	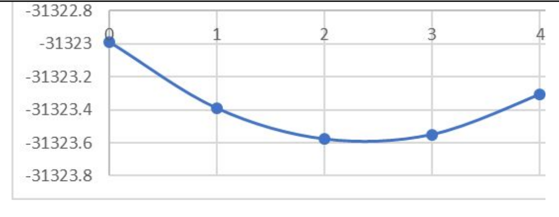
*CO	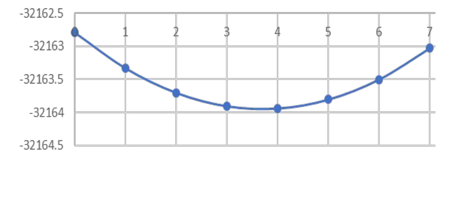	*CH_3	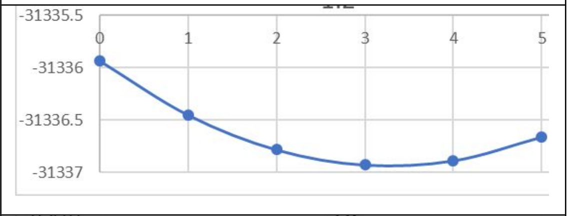
*CHO	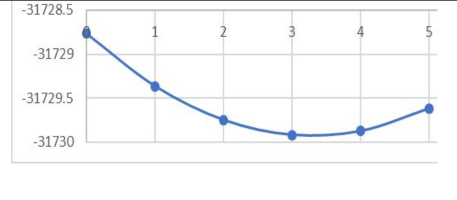	*CH_4	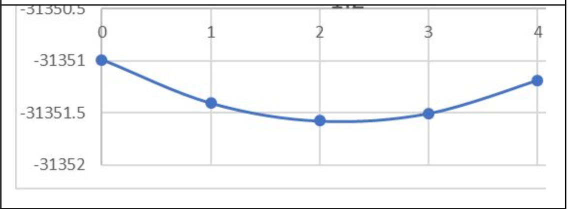

Table S2. Gibbs free energy vs. number of electrons profiles for the intermediates leading to ethylene, from *(CH)CO, obtained using the GCP-K method, at -1.2 V, pH=7 and T=298 K. Energies are reported in eV.

*(CH)CO	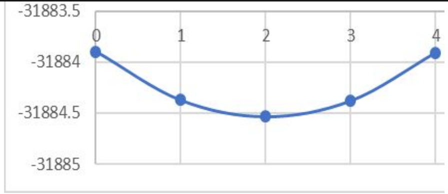	*(CH)COH	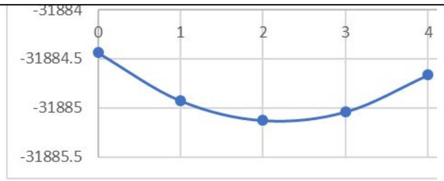
*(CH_2)CO	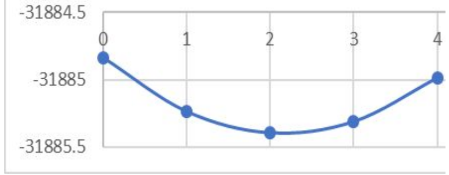	*CCH	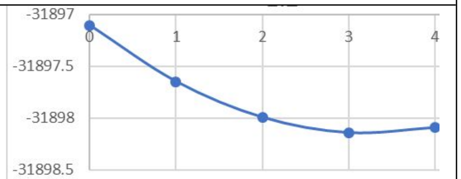
*(CH_2)COH	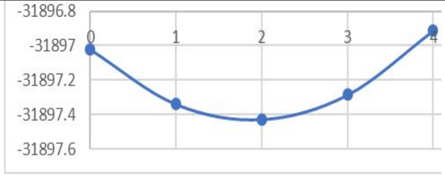	*CHCH	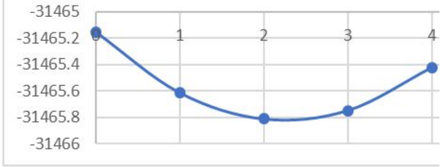
*CCH_2	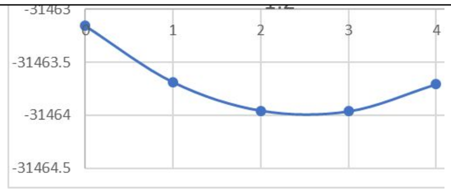	*$CHCH_2$	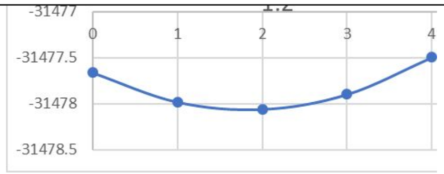
		*CH_2H_2	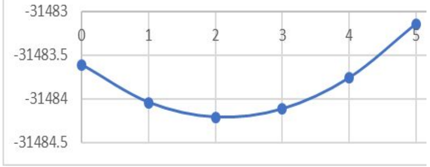

Table S3. Gibbs free energy vs. number of electrons profiles for the intermediates leading to ethylene, from *(CHO)CO, obtained using the GCP-K method, at -1.2 V, pH=7 and T=298 K. Energies are reported in eV.

*(CHO)CO	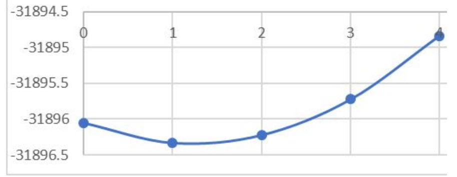	*CCH	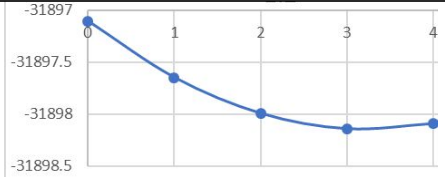
*(CHO)COH	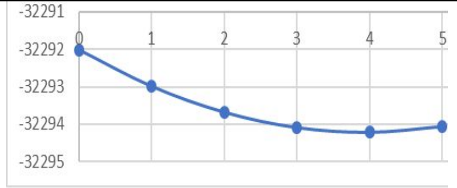	*CCH_2	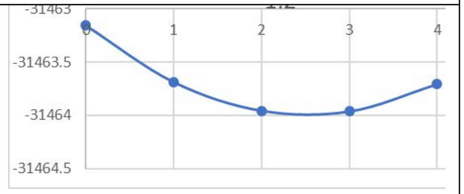
*(CHOH)COH	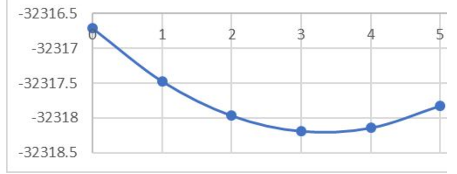	*CHCH	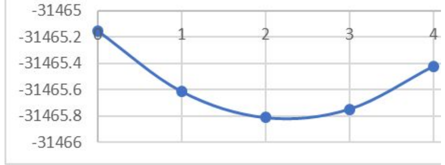
*CCHOH	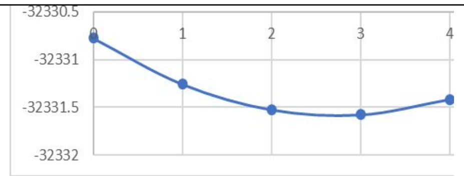	*$CHCH_2$	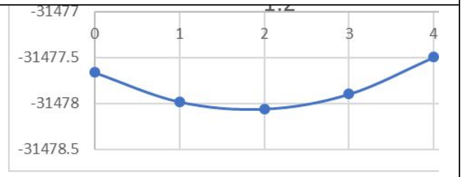
		*CH_2H_2	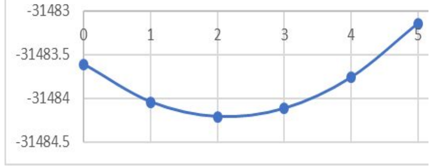

Table S4. Cu-C bond distances (in Angstrom) for the different intermediates at -1.2V. The C-N distances are reported in brackets.

*CO_2	2.38	*CHOH	1.84
*CO_2 bent	2.38	*CH	1.94 (1.39)
*HOCO	2.11	*CH_2	1.95 (1.41)
*CO	1.77	*CH_3	2.17
*CHO	1.92	*CH_4	3.34
*(CH)CO	1.98/1.90 (1.49/1.44)	*(CH)COH	1.91 (1.43)
*(CH_2)CO	2.31 (1.45/1.48)	*CCH	1.91/1.93 (1.45)
*(CH_2)COH	1.94 (1.48/1.43)	*CHCH	1.90 (1.43)
*CCH_2	1.98 (1.53/1.38)	*$CHCH_2$	1.96 (1.50/1.42)
*CH_2H_2	3.40	*(CHOH)COH	2.09
*(CHO)CO	1.94	*CCHOH	1.90 (1.44)
*(CHO)COH	2.00		

Table S5. Total spin of the system at neutral charge without external applied bias (U=0).

*CO_2	2.690	*CHOH	2.856
*CO_2 bent	2.690	*CH	2.570
*HOCO	2.835	*CH_2	2.649
*CO	2.849	*CH_3	2.541
*CHO	2.798	*CH_4	2.852
*(CH)CO	2.505	*(CH)COH	2.503
*(CH_2)CO	2.501	*CCH	2.676
*(CH_2)COH	2.491	*CHCH	2.502
*CCH_2	2.529	*$CHCH_2$	2.489
*CH_2H_2	2.848	*(CHOH)COH	2.836
*(CHO)CO	2.850	*CCHOH	2.725
*(CHO)COH	2.848		

Derivation of Grand Canonical Potential (GCP) parameters

The grand canonical potential kinetics (GCP-K) formulation derives the grand canonical potential (GCP) by Legendre transformation of canonical free energy, which is defined as,

$G(n;U) = F(n) - ne(U_{SHE} - U)$ (1)

We assume the canonical free energy quadratically depends on net charge.

$F(n) = a(n - n_0)^2 + b(n - n_0) + c$ (2)

Combining Eq. (1) and (2), we obtain the GCP,

$G(n,U) = a(n - n_0)^2 + b(n - n_0) + c - ne(U_{SHE} - U)$ (3)

For G(n,U) to be used as a thermodynamic potential, n must be equilibrated to U,

$dG(n,U)/dn = 0$ (4)

$n = -\frac{b - e(U_{SHE} - U)}{2a} + n_0$ (5)

$G(U) = - 1/4a(b - \mu_{e,SHE} - eU)^2 - n_0(\mu_{e,SHE} - eU) + c$ (6)

The number of electrons at potential U is thus:

$n(U) = -\frac{1 \partial GCP(U)}{e \ \partial U} = n_0 - \frac{1}{2ae}(b - \mu_{e,SHE} + eU)$ (7)

b can be determined using $n(U_{PZC}) = n_0$ which leads to

$b = \mu_{e,SHE} - eU_{PZC}$ (8)

From these calculations we can obtain the differential capacitance (C_{diff}).

$C_{diff} = \partial n/\partial U = -1/2a$ (9)

The C_{diff} and Potential of Zero Charge (PZC) for various cases are tabulated in Table S6.

Table S6. GCP parameters for all investigated intermediates.

System	a [V/e]	b [V]	c [eV]	C_{diff} [F]	PZC [V vs. SHE]
Cu-MOF	0.1612960	-3.733774	-30331.43	3.09989	-0.92623
*CO_2	0.1625973	-3.474706	-33254.20	-3.07508	-1.18529
*CO_2 bent	0.1251544	-3.806234	-33253.61	-3.99507	-0.85377
*HOCO	0.1497261	-3.924300	-33253.33	-3.33943	-0.7357
*CO	0.0842745	-3.669624	-33269.66	-5.93299	-0.99038
*CHO	0.1073923	-3.753306	-32815.03	-4.65583	-0.90669
*CHOH	0.1006394	-3.714057	-32829.50	-4.96823	-0.94594
*CH	0.1485439	-3.916815	-32845.49	-3.36601	-0.74319
*CH_2	0.1076085	-3.556376	-32393.21	-4.64647	-1.10362
*CH_3	0.0928229	-3.654847	-32409.14	-5.3866	-1.00515
*CH_4	0.1216861	-3.581959	-32426.53	-4.10893	-1.07804
*(CH)CO	0.15734	-3.678639	-32984.41	-3.17783	-0.98136
*(CH_2)CO	0.120157	-3.565093	-32985.15	-4.16122	-1.09491
*(CH_2)COH	0.115108	-3.479548	-32999.68	-4.34375	-1.18045
*CCH_2	0.131229	-3.708863	-32545.41	-3.81013	-0.95114
*CH_2H_2	0.131408	-3.609673	-32583.32	-3.80494	-1.05033
*(CHO)CO	0.070587	-3.531669	-33422.73	-7.08346	-1.12833
*(CHO)COH	0.140613	-4.157215	-33422.19	-3.55586	-0.50279
*(CH)COH	0.143732	-3.677756	-32984.62	-3.4787	-0.98224
*CCH	0.099205	-3.690499	-33000.09	-5.04007	-0.9695
*CHCH	0.130775	-3.637045	-32547.33	-3.82336	-1.02296
*$CHCH_2$	0.120819	-3.487983	-32562.27	-4.13842	-1.17202
*(CHOH)COH	0.134868	-3.941867	-33437.90	-3.70733	-0.71813
*CCHOH	0.106615	-3.632075	-33454.12	-4.68977	-1.02793

Table S7. CH_4 and C_2H_4 selectivity using various electrocatalysts for CO2RR reported in literature.

Catalyst	Electrolyte	Potential (V)	Main product (FE/%)	Partial current density (mA cm^{-2})	Stability / h	Ref.
Cu nanoparticles deposited GDE	1 M KOH	-2.0 (*vs.* Ag/AgCl)	C_2H_4 (~50)CO (~33) H_2 (~10) C_2H_5OH (~5) CH_4 (~5)	~50	--	1
Cu-Al	1 M KOH	-1.8 (*vs.* RHE)	C_2H_4 (~80)	600	5	2
Densely packed Cu particle	0.1 M $KHCO_3$	-0.86 (*vs.* RHE)	C_2H_4 (~34) C_2H_5OH (~15)CH_4 (~5)	--	10	3
Cu mesocrystals	0.1 M $KHCO_3$	-0.99 (*vs.* RHE)	C_2H_4 (27.2) CH_4 (1.47)H_2 (~60)	--	~6	4
44 nm Cu nanocube	0.1 M $KHCO_3$	-1.1 (*vs.* RHE)	C_2H_4 (~40) CH_4 (~20)H_2 (~20)	--	1	5
Phase-separated CuPd	1 M KOH	-0.74 (*vs.* RHE)	C_2H_4 (~47) CO (~18) C_2H_5OH (~15)	360.5	--	6
CuO/N_x C-700 °C	0.1 M $NaHCO_3$	-1.25 (*vs.* RHE)	C_2H_4 (36) CH_4 (~14)CO (~12) H_2 (30)	--	~1	7
Cu nanocube-O	0.1 M $KHCO_3$	-1.0 (*vs.* RHE)	C_2H_4 (45) C_2H_5OH (~22.5)	~15	0.25-0.3	8
KF cycled Cu foil	0.1 M $KHCO_3$	-1.0 (*vs.* RHE)	C_2H_4 (16.3) C_2H_5OH (7.9) H_2 (~58)	--	--	9
8.1 µm Cu nanowire	0.1 M $KHCO_3$	-1.1 (*vs.* RHE)	C_2H_4 (17.4) C_2H_5OH (~5) C_2H_6 (2) CO (~10) n-propanol (~10)HCOOH (~15)	--	5	10
Polycrystalline Cu with N-tolylpyridinium chloride	0.1 M $KHCO_3$	-1.1 (*vs.* RHE)	C_2H_4 (~40) C_2H_5OH (~30) 1-propanol (~7)HCOOH (~6)	~0.13	10	11
CuAg	1 M KOH	-0.7 (*vs.* RHE)	C_2H_4 (~55) C_2H_5OH (~25)CO (~7)	~170	--	12

PcCu-Cu-O	0.1 M $KHCO_3$	-1.2 (*vs*. RHE)	C_2H_4 (50)	7.3	4	13
Cu nanoparticle	1 M KOH	-0.8 (*vs*. RHE)	C_2H_4 (~45) C_2H_5OH (~17.5)H_2 (~7) CO (~20)	~140	4	14
Cu_4Zn	0.1 M $KHCO_3$	-1.05 (*vs*. RHE)	C_2H_4 (~10) C_2H_5OH (~30)CO (~10)	--	10	15
Single-atomic Cu-substituted CeO_2	0.1 M $KHCO_3$	-1.8 (*vs*. RHE)	C_2H_4 (~15) CH_4 (58) H_2 (~20)	70	2.2	16
Cu/MoS_2	0.1 M $NaHCO_3$	-1.4 (*vs*. Ag/AgCl)	C_2H_4 (2.93) CH_4 (17.08) H_2 (33.07) CO (35.19)	~4	48	17
Polycrystalline Cu	0.1 M $NaHCO_3$	-1.6 (*vs*. Ag/AgCl)	CH_4 (50) H_2 (~20) C_2H_4 (10)	~4	1	18
Cu nanoparticle	0.1 M $KHCO_3$	-1.1 (*vs*. RHE)	CH_4 (~20) H_2 (~43) C_2H_4 (~15)	~1.1	--	19
Cu foil	0.1 M $KHCO_3$	-1.1 (*vs*. RHE)	CH_4 (57) H_2 (~20) C_2H_4 (~20)	23	--	20
Polished Cu foil	0.3 M KI + 0.1 M $KHCO_3$	-1.0 (*vs*. RHE)	CH_4 (~56) H_2 (~12) C_2H_4 (~23) HCOOH (~5)	--	0.17	21
Cu nanocube	0.1 M $KHCO_3$	-1.05 (*vs*. RHE)	CH_4 (~56) CO (~5) C_2H_4 (~20)	--	~5	22
Cu_2O film	0.1 M $KHCO_3$	-0.99 (*vs*. Ag/AgCl)	CH_4 (9.85) H_2 (26.21) C_2H_4 (32.92) $HCOO^-$ (12.67)	1	--	23
CuO_x nanoparticle	0.1 M $KHCO_3$	-1.0 (*vs*. RHE)	CH_4 (~19) C_2H_4	~1.3	6.7	24

			(~21)			
Cu dimer distorted HKUST-1	1 M KOH	-1.07 (*vs.* RHE)	C_2H_4 (45) H_2 (7) CH_4 (0.4) CO (24)	262	7.5	25
Cu_2O@HKUST-1	0.1 M $KHCO_3$	-1.71 (*vs.* RHE)	CH_4 (63.2) C_2H_4 (16.2)	8.4	1	26
copper(II)-5,10,15,20-tetrakis(2,6-dihydroxyphenyl)porphyrin	0.5 M $KHCO_3$	-0.976 (*vs.* RHE)	CH_4 (~27) C_2H_4 (~17) CO (~10)	13.2	~1	27
Reconstructed Cu	0.05 M $KHCO_3$	~-2.0 (*vs.* RHE)	C_2H_4 (~56)	~18	--	28
Cu-ade MOF	0.1 M $KHCO_3$	-1.4 (*vs.* RHE)	C_2H_4 (45)	8.5	8	29
HATNA-Cu-MOF powder	0.1 M $KHCO_3$		CH_4 (~50)	7.4	12	30

References

1. Song, H.; Song, J. T.; Kim, B.; Tan, Y. C.; Oh, J., Activation of C_2H_4 reaction pathways in electrochemical CO_2 reduction under low CO_2 partial pressure. *Appl. Catal. B-Environ.* **2020**, *272*, 119049.

2. Bao, H.; Qiu, Y.; Peng, X.; Wang, J. A.; Mi, Y.; Zhao, S.; Liu, X.; Liu, Y.; Cao, R.; Zhuo, L.; Ren, J.; Sun, J.; Luo, J.; Sun, X., Isolated copper single sites for high-performance electroreduction of carbon monoxide to multicarbon products. *Nat. Commun.* **2021**, *12*, 238.

3. Kim, D.; Kley, C. S.; Li, Y.; Yang, P., Copper nanoparticle ensembles for selective electroreduction of CO_2 to C2–C3 products. *Proc. Natl. Acad. Sci. USA* **2017**, *114*, 10560-10565.

4. Chen, C. S.; Handoko, A. D.; Wan, J. H.; Ma, L.; Ren, D.; Yeo, B. S., Stable and selective electrochemical reduction of carbon dioxide to ethylene on copper mesocrystals. *Catal. Sci. Technol.* **2015**, *5*, 161-168.

5. Loiudice, A.; Lobaccaro, P.; Kamali, E. A.; Thao, T.; Huang, B. H.; Ager, J. W.; Buonsanti, R., Tailoring copper nanocrystals towards C2 products in electrochemical CO_2 reduction. *Angew. Chem. Int. Ed.* **2016**, *55*, 5789-5792.

6. Ma, S.; Sadakiyo, M.; Heima, M.; Luo, R.; Haasch, R. T.; Gold, J. I.; Yamauchi, M.; Kenis, P. J. A., Electroreduction of carbon dioxide to hydrocarbons using bimetallic Cu–Pd catalysts with different mixing patterns. *J. Am. Chem. Soc.* **2017**, *139*, 47-50.
7. Yang, H.-J.; Yang, H.; Hong, Y.-H.; Zhang, P.-Y.; Wang, T.; Chen, L.-N.; Zhang, F.-Y.; Wu, Q.-H.; Tian, N.; Zhou, Z.-Y.; Sun, S.-G., Promoting ethylene selectivity from CO_2 electroreduction on CuO supported onto CO_2 capture materials. *Chem. Sus. Chem.* **2018**, *11*, 881-887.
8. Gao, D.; Zegkinoglou, I.; Divins, N. J.; Scholten, F.; Sinev, I.; Grosse, P.; Roldan Cuenya, B., Plasma-activated copper nanocube catalysts for efficient carbon dioxide electroreduction to hydrocarbons and alcohols. *ACS Nano* **2017**, *11*, 4825-4831.
9. Kwon, Y.; Lum, Y.; Clark, E. L.; Ager, J. W.; Bell, A. T., CO_2 electroreduction with enhanced ethylene and ethanol selectivity by nanostructuring polycrystalline copper. *Chem. Electro. Chem.* **2016**, *3*, 1012-1019.
10. Ma, M.; Djanashvili, K.; Smith, W. A., controllable hydrocarbon formation from the electrochemical reduction of CO_2 over Cu nanowire arrays. *Angew. Chem. Int. Ed.* **2016**, *55*, 6680-6684.
11. Han, Z.; Kortlever, R.; Chen, H.-Y.; Peters, J. C.; Agapie, T., CO_2 reduction selective for $C\geq2$ products on polycrystalline copper with N-substituted pyridinium additives. *ACS Cent. Sci.* **2017**, *3*, 853-859.
12. Hoang, T. T. H.; Verma, S.; Ma, S.; Fister, T. T.; Timoshenko, J.; Frenkel, A. I.; Kenis, P. J. A.; Gewirth, A. A., Nanoporous Copper–silver alloys by additive-controlled electrodeposition for the selective electroreduction of CO_2 to ethylene and ethanol. *J. Am. Chem. Soc.* **2018**, *140*, 5791-5797.
13. Qiu, X.-F., Zhu, H.-L., Huang, J.-R., Liao, P.-Q., Chen, X.-M., Highly selective CO_2 electroreduction to C_2H_4 using a metal−organic framework with dual active sites. *J. Am. Chem. Soc.* **2021**, *143*, 7242−7246.
14. Ma, S.; Sadakiyo, M.; Luo, R.; Heima, M.; Yamauchi, M.; Kenis, P. J. A., One-step electrosynthesis of ethylene and ethanol from CO_2 in an alkaline electrolyzer. *J. Power Sources* **2016**, *301*, 219-228.
15. Ren, D.; Ang, B. S.-H.; Yeo, B. S., Tuning the selectivity of carbon dioxide electroreduction toward ethanol on oxide-derived CuxZn catalysts. *ACS Catal.* **2016**, *6*, 8239-8247.

16. Wang, Y.; Chen, Z.; Han, P.; Du, Y.; Gu, Z.; Xu, X.; Zheng, G., Single-atomic Cu with multiple oxygen vacancies on ceria for electrocatalytic CO_2 reduction to CH_4. *ACS Catal.* **2018**, *8*, 7113-7119.

17. Shi, G.; Yu, L.; Ba, X.; Zhang, X.; Zhou, J.; Yu, Y., Copper nanoparticle interspersed MoS_2 nanoflowers with enhanced efficiency for CO_2 electrochemical reduction to fuel. *Dalton Trans.* **2017**, *46*, 10569-10577.

18. Ahn, S. T.; Abu-Baker, I.; Palmore, G. T. R., Electroreduction of CO_2 on polycrystalline copper: Effect of temperature on product selectivity. *Catal. Today* **2017**, *288*, 24-29.

19. Resasco, J.; Chen, L. D.; Clark, E.; Tsai, C.; Hahn, C.; Jaramillo, T. F.; Chan, K.; Bell, A. T., Promoter effects of alkali metal cations on the electrochemical reduction of carbon dioxide. *J. Am. Chem. Soc.* **2017**, *139*, 11277-11287.

20. Reske, R.; Mistry, H.; Behafarid, F.; Roldan Cuenya, B.; Strasser, P., Particle size effects in the catalytic electroreduction of CO_2 on Cu nanoparticles. *J. Am. Chem. Soc.* **2014**, *136*, 6978-6986.

21. Varela, A. S.; Ju, W.; Reier, T.; Strasser, P., Tuning the catalytic activity and selectivity of Cu for CO_2 electroreduction in the presence of halides. *ACS Catal.* **2016**, *6*, 2136-2144.

22. Grosse, P.; Gao, D.; Scholten, F.; Sinev, I.; Mistry, H.; Roldan Cuenya, B., Dynamic changes in the structure, chemical state and catalytic selectivity of Cu nanocubes during CO_2 electroreduction: size and support effects. *Angew. Chem. Int. Ed.* **2018**, *57*, 6192-6197.

23. Ren, D.; Deng, Y.; Handoko, A. D.; Chen, C. S.; Malkhandi, S.; Yeo, B. S., Selective electrochemical reduction of carbon dioxide to ethylene and ethanol on copper(I) oxide catalysts. *ACS Catal.* **2015**, *5*, 2814-2821.

24. Wang, X.; Varela, A. S.; Bergmann, A.; Kühl, S.; Strasser, P., Catalyst particle density controls hydrocarbon product selectivity in CO_2 electroreduction on CuOx. *Chem. Sus. Chem.* **2017**, *10*, 4642-4649.

25. Nam, D. H.; Bushuyev, O. S.; Li, J.; De Luna, P.; Seifitokaldani, A.; Dinh, C. T.; Garcia de Arquer, F. P.; Wang, Y.; Liang, Z.; Proppe, A. H.; Tan, C. S.; Todorovic, P.; Shekhah, O.; Gabardo, C. M.; Jo, J. W.; Choi, J.; Choi, M. J.; Baek, S. W.; Kim, J.; Sinton, D.; Kelley, S. O.; Eddaoudi, M.; Sargent, E. H., Metal-organic frameworks mediate Cu coordination for selective CO_2 electroreduction. *J. Am. Chem. Soc.* **2018**, *140*, 11378-11386.

26. Tan, X.; Yu, C.; Zhao, C.; Huang, H.; Yao, X.; Han, X.; Guo, W.; Cui, S.; Huang, H.; Qiu, J., Restructuring of Cu_2O to Cu_2O@Cu-metal–organic frameworks for selective electrochemical reduction of CO_2. *ACS Appl. Mater. Interfaces* **2019**, *11*, 9904-9910.
27. Weng, Z.; Jiang, J.; Wu, Y.; Wu, Z.; Guo, X.; Materna, K. L.; Liu, W.; Batista, V. S.; Brudvig, G. W.; Wang, H., Electrochemical CO_2 reduction to hydrocarbons on a heterogeneous molecular Cu catalyst in aqueous solution. *J. Am. Chem. Soc.* **2016**, *138*, 8076-8079.
28. Kibria, M. G.; Dinh, C. T.; Seifitokaldani, A.; De Luna, P.; Burdyny, T.; Quintero-Bermudez, R.; Ross, M. B.; Bushuyev, O. S.; Garcia de Arquer, F. P.; Yang, P.; Sinton, D.; Sargent, E. H., A surface reconstruction route to high productivity and selectivity in CO_2 electroreduction toward C2+ hydrocarbons. *Adv. Mater.* **2018**, *30*, 1804867.
29. Yang, F.; Chen, A.; Deng, P. L.; Zhou, Y.; Shahid, Z.; Liu, H.; Xia, B. Y., Highly efficient electroconversion of carbon dioxide into hydrocarbons by cathodized copper–organic frameworks. *Chem. Sci.* **2019**, *10*, 7975-7981.
30. Liu, Y.; Li, S.; Dai, L.; Li, J.; Lv, J.; Zhu, Z.; Yin, A.; Li, P. F.; Wang, B. The synthesis of hexaazatrinaphthylene based 2D conjugated copper metal-organic framework for highly selective and stable electroreduction of CO_2 to methane. *Angew. Chem. Int. Ed.* **2021**, *60*, 16409−16415.
